# The Prevalence of Obesity Among Children With Type 2 Diabetes: A Systematic Review and Meta-analysis

**DOI:** 10.1001/jamanetworkopen.2022.47186

**Published:** 2022-12-15

**Authors:** Milena Cioana, Jiawen Deng, Ajantha Nadarajah, Maggie Hou, Yuan Qiu, Sondra Song Jie Chen, Angelica Rivas, Laura Banfield, Parm Pal Toor, Fangwen Zhou, Ayla Guven, Haifa Alfaraidi, Ahlam Alotaibi, Lehana Thabane, M. Constantine Samaan

**Affiliations:** 1 Department of Pediatrics, McMaster University, Hamilton, Ontario, Canada; 2 Division of Pediatric Endocrinology, McMaster Children’s Hospital, Hamilton, Ontario, Canada; 3 Michael G. De Groote School of Medicine, McMaster University, Hamilton, Ontario, Canada; 4 Health Sciences Library, McMaster University, Hamilton, Ontario, Canada; 5 Health Science University, Zeynep Kamil Maternity and Children Hospital, Pediatric Endocrinology Clinic, Istanbul, Turkey; 6 College of Medicine, King Saud bin Abdulaziz University for Health Sciences, Division of Endocrinology, Department of Pediatrics, Ministry of the National Guard Health Affairs, Riyadh, Saudi Arabia; 7 Division of Pediatric Endocrinology, Department of Pediatrics, King Abdullah bin Abdulaziz University Hospital, Princess Noura University, Riyadh, Saudi Arabia; 8 Department of Health Research Methods, Evidence and Impact, McMaster University, Hamilton, Ontario, Canada; 9 Department of Anesthesia, McMaster University, Hamilton, Ontario, Canada; 10 Centre for Evaluation of Medicines, St Joseph’s Healthcare, Hamilton, Ontario, Canada; 11 Biostatistics Unit, St Joseph’s Healthcare, Hamilton, Ontario, Canada

## Abstract

**Importance:**

The childhood obesity epidemic is presumed to drive pediatric type 2 diabetes (T2D); however, the global scale of obesity in children with T2D is unknown.

**Objectives:**

To evaluate the global prevalence of obesity in pediatric T2D, examine the association of sex and race with obesity risk, and assess the association of obesity with glycemic control and dyslipidemia.

**Data Sources:**

MEDLINE, Embase, CINAHL, Cochrane Library, and Web of Science were searched from database inception to June 16, 2022.

**Study Selection:**

Observational studies with at least 10 participants reporting the prevalence of obesity in patients with pediatric T2D were included.

**Data Extraction and Synthesis:**

Following the Meta-analysis of Observational Studies in Epidemiology reporting guideline, 2 independent reviewers in teams performed data extraction and risk of bias and level of evidence analyses. The meta-analysis was conducted using a random-effects model.

**Main Outcomes and Measures:**

The primary outcomes included the pooled prevalence rates of obesity in children with T2D. The secondary outcomes assessed pooled prevalence rates by sex and race and associations between obesity and glycemic control and dyslipidemia.

**Results:**

Of 57 articles included in the systematic review, 53 articles, with 8942 participants, were included in the meta-analysis. The overall prevalence of obesity among pediatric patients with T2D was 75.27% (95% CI, 70.47%-79.78%), and the prevalence of obesity at diabetes diagnosis among 4688 participants was 77.24% (95% CI, 70.55%-83.34%). While male participants had higher odds of obesity than female participants (odds ratio, 2.10; 95% CI, 1.33-3.31), Asian participants had the lowest prevalence of obesity (64.50%; 95% CI, 53.28%-74.99%), and White participants had the highest prevalence of obesity (89.86%; 95% CI, 71.50%-99.74%) compared with other racial groups. High heterogeneity across studies and varying degrees of glycemic control and dyslipidemia were noted.

**Conclusions and Relevance:**

The findings of this systematic review and meta-analysis suggest that obesity is not a universal phenotype in children with T2D. Further studies are needed to consider the role of obesity and other mechanisms in diabetes genesis in this population.

## Key Points

**Question:**

What is the prevalence of obesity in pediatric patients with type 2 diabetes (T2D)?

**Findings:**

This systematic review and meta-analysis of 53 studies including 8942 participants found that 75.27% of children with T2D had obesity, and 77.24% had obesity at diagnosis. Male participants had significantly higher odds of obesity than female participants, and Asian participants had the lowest prevalence of obesity compared with other racial groups.

**Meaning:**

In this study, not all pediatric patients with T2D had obesity; further studies are needed to elucidate the mechanisms beyond obesity driving this condition in children.

## Teaser

This systematic review and meta-analysis evaluates the global prevalence of obesity in pediatric type 2 diabetes, examines the association of sex and race with obesity risk, and assesses the association of obesity with glycemic control and dyslipidemia.

## Introduction

In the past few decades, type 2 diabetes (T2D) in children and adolescents has emerged in conjunction with increasing pediatric obesity rates globally.^1,2,3,4,5,6,7,8^ Children and youth living with obesity also have a higher risk of developing T2D as adults when compared with children with reference range weight, which may contribute to increased cardiovascular risk.^9^

T2D is an aggressive disease in children with high treatment failure rates. It has early comorbidities and complications, including nonalcoholic fatty liver disease, dyslipidemia, polycystic ovary syndrome, and nephropathy.^9,10,11,12,13,14,15,16^

While the complex weave of factors driving the pathogenesis of pediatric T2D are not yet fully defined,^17,18,19^ the biopsychosocial determinants of health with health inequities and social and economic vulnerabilities in this population play an important role in disease risk and outcomes.^20^ Obesity is a major trigger for screening for T2D in clinical practice,^21,22,23^ yet the prevalence of obesity in the pediatric T2D population is unknown. It is important to recognize whether T2D is diagnosed through ascertainment bias, whereby only children with obesity are screened and subsequently diagnosed with T2D. If obesity is not a universal phenotype in T2D, there may be children with reference range body mass measures in whom T2D is driven by factors other than obesity, which impacts their treatment and outcomes. Estimating the prevalence of obesity in the pediatric T2D population may have a significant impact on the recommendations of screening guidelines for the disease.

This systematic review and meta-analysis aimed to evaluate the global prevalence of obesity in children and adolescents living with T2D and assess the association of sex and race with obesity prevalence in this population. Furthermore, we explored the association of obesity with T2D-related metabolic profiles, including glycemic control and lipid homeostasis.

## Methods

### Protocol and Registration

This systematic review was registered with the International Prospective Register of Systematic Reviews (CRD42018091127).^24^ This review is reported as per the Meta-analysis of Observational Studies in Epidemiology (MOOSE) checklist.^25^

### Eligibility Criteria

Primary observational studies (cross-sectional, retrospective cohort, or prospective cohort) with a sample size of at least 10 participants reporting the prevalence of obesity in children 18 years or younger with T2D were included. T2D diagnostic criteria were (1) random plasma glucose of at least 200.0 mg/dL and presence of classical symptoms (to convert glucose to millimoles per liter, multiply by 0.0555), (2) fasting plasma glucose of at least 127.9 mg/dL, or (3) 2-hour plasma glucose of at least 200.0 mg/dL in response to oral glucose tolerance test and the absence of pancreatic autoantibodies.^21,22,23^ We incorporated studies that used age- and sex- adjusted body mass index (BMI)–based measures to define overweight and obesity, with BMIs in the 85th percentile or greater to less than the 95th percentile defining overweight and BMIs in the 95th percentile or greater defining obesity.^26^ Studies with different definitions of obesity were included but removed in the sensitivity analysis to assess their impact on the results.

We excluded studies reporting on participants with gestational diabetes. If study reports involved serial data publication, we included the report with the largest sample size.

### Literature Searches

The literature searches encompassed journal articles, conference abstracts, and gray literature. No language- or time-based restrictions were applied, but the searches were restricted to human studies.

Search strategies were developed by a senior health sciences librarian and conducted in MEDLINE, Embase, Cumulative Index of Nursing and Allied Health Literature (CINAHL), Cochrane Central Register of Controlled Trials, and Cochrane Database of Systematic Reviews from the date of inception (eTables 1-5 in Supplement 1). The gray literature searches were conducted in clinicaltrials.gov and Web of Science: Conference Proceedings Citation Index—Science. The initial search was performed on December 14, 2017; updated searches were conducted on February 1, 2019, and June 16, 2022.

In addition, we searched the references of the articles screened for full-text eligibility to retrieve studies for inclusion. We searched for full-text publications where conference abstracts were eligible; if not located, we contacted the principal investigators to determine publication status and obtain relevant data for the analyses.

### Study Selection and Data Collection

Two independent reviewers in pairs (M.C., J.D., A.N., M.H., Y.Q., S.S.J.C., A.R., P.P.T., and F.Z.) screened titles, abstracts, and full-text articles and completed data abstraction. Reviewers resolved any differences at all data assessment stages through discussions, and a third reviewer (M.C.S.) resolved persistent disagreements. A standardized data abstraction form was developed and piloted specifically for this study. The data collected included the authors’ names; title; year of publication; country; study design; age at T2D diagnosis; age at study enrollment; diabetes duration; sample size; prevalence of reference-range weight, overweight, and obesity; and hemoglobin A_1c_ (HbA_1c_) values to assess glycemic control. We also collected data on participants’ lipid profiles to assess for dyslipidemia, including triglycerides, total cholesterol, low-density lipoprotein (LDL) cholesterol, and high-density lipoprotein (HDL) cholesterol. In addition, we extracted the sex, race and ethnicity, and specific obesity prevalence data when available. If longitudinal studies reported obesity prevalence at multiple time points, we abstracted the data closest to the date of T2D diagnosis. We contacted the principal investigators to retrieve missing data.

### Risk of Bias and Level of Evidence Assessment

Two independent reviewers (M.C. and J.D.) assessed the risk of bias using a validated tool developed by Hoy et al.^27^ A third reviewer (M.C.S.) arbitrated persistent disagreements. The overall level of evidence was assessed according to the Oxford Centre for Evidence-Based Medicine criteria.^28^

### Statistical Analysis

A random-effects meta-analysis was performed when 2 or more eligible studies of similar design, methods, populations, and outcomes were identified.^29,30^ The primary outcome for this systematic review was assessing the overall pooled global prevalence of obesity in T2D. Because studies in which prevalence trended toward 0% or 100% may affect the meta-analysis, each study’s prevalence values were transformed using the Freeman-Tukey double arcsine method, and the results were then converted back to prevalence estimates for interpretation.^30^

Both inconsistency index (*I*^2^) and χ^2^ test *P* values were used to quantify heterogeneity. An *I*^2^ value of greater than 75% and 1-sided *P* < .10 were considered significant.^31^

Subgroup analyses were performed by sex and race, if data were available.^5,13,32^ When 2 or more studies reported the prevalence of obesity, we evaluated a pooled prevalence for each sex and pooled odds ratio (OR). We used the National Institutes of Health definitions of racial and ethnic groups to categorize the included studies and used the term Indigenous to refer to Indigenous populations in North America.^33^

Metaregression analyses were performed to examine the associations between the prevalence of obesity and mean HbA_1c_ as a measure of glycemic control and dyslipidemia. In addition, we conducted sensitivity analyses by removing conference abstracts with no associated full-text publications, sample sizes smaller than 50, studies with mixed ages when pediatric-only data could not be obtained, and those that used different obesity definitions. Subgroup and sensitivity analyses were to be conducted if at least 10 studies were included in the meta-analysis for the specific outcomes.^31^ We also performed post hoc sensitivity analyses by excluding studies with inclusion criteria of overweight, those with unspecified or unclear diabetes diagnostic criteria, those with patients who had weight loss at presentation or positive pancreatic autoantibodies, those that did not explicitly assess for and exclude maturity-onset diabetes of the young (MODY), and those that were not population based.

A contour-enhanced funnel plot was used to investigate publication bias. The Egger test and visual inspection were used to assess plot asymmetry.^31^

The meta-analysis and forest plots were generated using the metafor package in RStudio, version 1.1.383, using the R language version 3.4.3 (R Project for Statistical Computing).^34,35,36^ The forest plots for the OR by sex meta-analysis were generated using Review Manager version 5.3 software.^37^

## Results

### Study Selection

The study screening and selection process is illustrated in the flow diagram (eFigure 1 in Supplement 1). We screened 13 449 nonduplicated records, and 57 studies from unique populations were included in the review. Fifty-three studies, with 8942 participants, were included in our meta-analysis.

### Study Characteristics

Overall, 26 studies (45.60%) had a cross-sectional design,^38,39,40,41,42,43,44,45,46,47,48,49,50,51,52,53,54,55,56,57,58,59,60,61,62,63^ 23 (40.40%) were retrospective cohort studies,^64,65,66,67,68,69,70,71,72,73,74,75,76,77,78,79,80,81,82,83,84,85,86^ and 8 (14.00%) were prospective cohort studies.^3,11,87,88,89,90,91,92^ eTable 6 in Supplement 1 reports the characteristics of the included studies.

Of the 57 studies, 12 did not report specific diabetes diagnostic criteria,^43,49,59,64,69,70,72,75,76,78,84,91^ and 18 did not report measuring autoantibodies.^44,49,58,59,62,65,69,70,73,74,75,76,77,78,79,83,84,85^ Of the studies reporting autoantibody testing results, 55 patients had positive tests.

The most common clinical presentations included acanthosis nigricans, polyuria, and polydipsia. The most commonly reported risk factors included family history of T2D and maternal gestational diabetes. Most patients were treated with oral hypoglycemic agents, and some were treated with insulin, diet alone, or combination therapies (eTable 7 in Supplement 1).

Several studies did not separate the diagnosis by age and included participants older than 18 years, highlighting the different definitions of pediatric age groups globally. We included some of these studies as long as most study participants were younger than 18 years.

### Pooled Prevalence of Obesity

**Figure 1.**
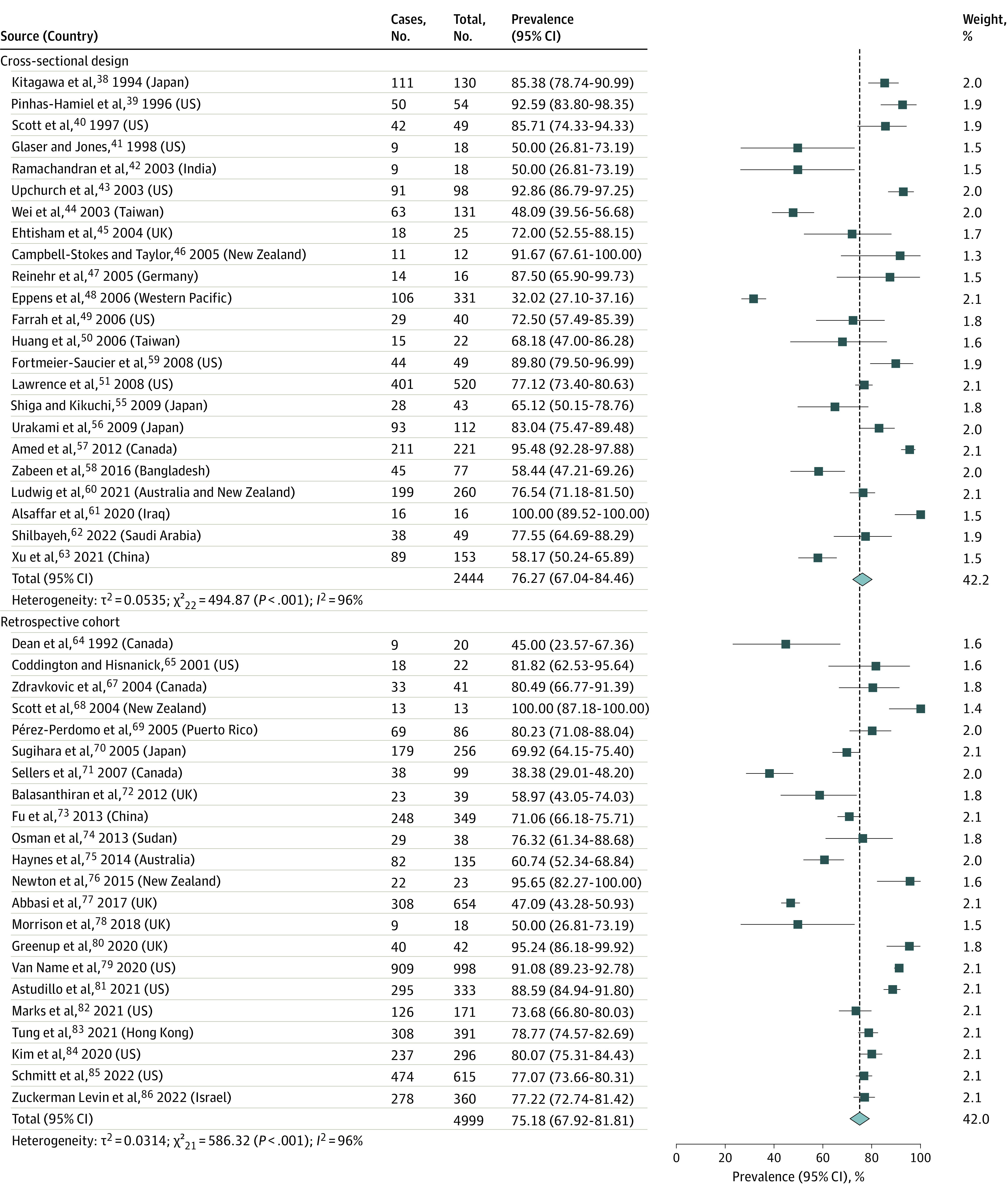
Pooled Obesity Prevalence in Cross-sectional and Retrospective Cohort Studies of Pediatric Type 2 Diabetes, by Study Design

**Figure 2.**
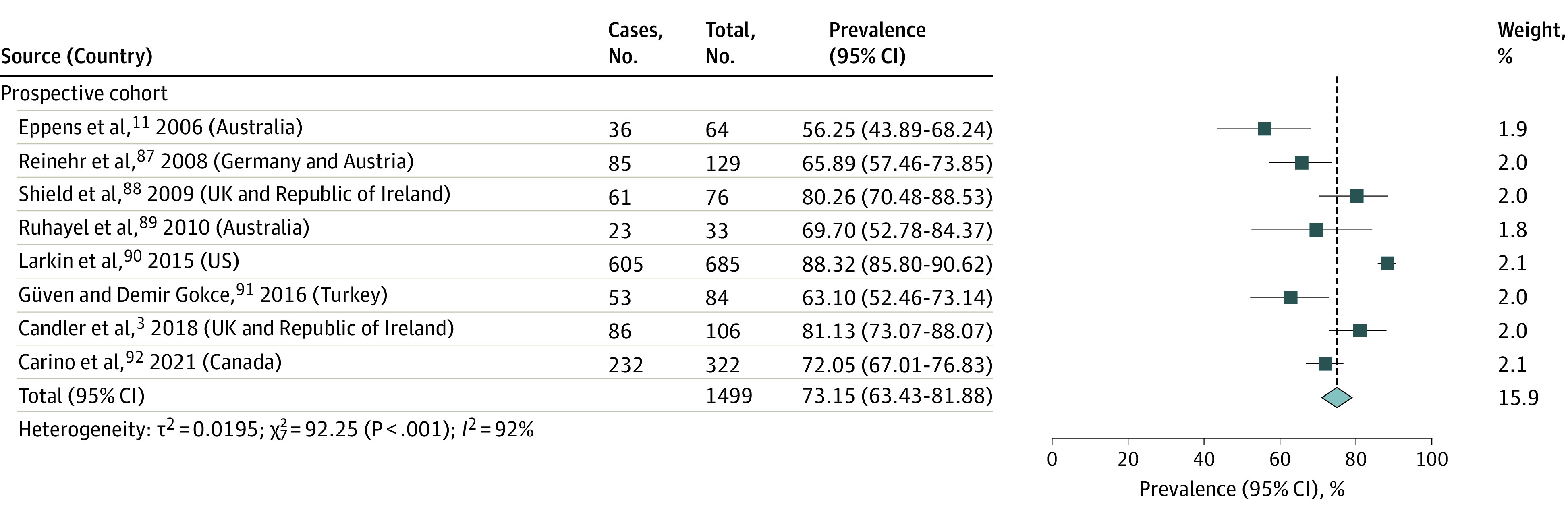
Pooled Obesity Prevalence in Prospective Cohort Studies of Pediatric Type 2 Diabetes

Data from 53 studies with 8942 participants estimated the overall pooled prevalence of obesity in pediatric patients with T2D to be 75.27% (95% CI, 70.47%-79.78%; *I*^2^ = 96%; *P* < .001) (Figure 1 and Figure 2).^3,11,38,39,40,41,42,43,44,45,46,47,48,49,50,51,55,56,57,58,59,60,61,62,63,64,65,67,68,69,70,71,72,73,74,75,76,77,78,79,80,81,82,83,84,85,86,87,88,89,90,91,92^ Four of the 57 studies were not included in this meta-analysis, with 1 study not providing an exact prevalence estimate,^66^ and 3 containing race-based subgroup data already reported in another article.^51,52,53,54^ Obesity prevalence was similar across study designs (cross-sectional studies^38,39,40,41,42,43,44,45,46,47,48,49,50,51,55,56,57,58,59,60,61,62,63^: 76.27%; 95% CI, 67.04%-84.46%; *I*^2^ = 96%; *P* < .001; n = 2444; retrospective cohort studies^64,65,67,68,69,70,71,72,73,74,75,76,77,78,79,80,81,82,83,84,85,86^: 75.18%; 95% CI, 67.92%-81.81%; *I*^2^ = 96%; *P* < .001; n = 4999; prospective cohort studies^3,11,87,88,89,90,91,92^: 73.15%; 95% CI, 63.43%-81.88%; *I*^2^ = 92%; *P* < .001; n = 1499) (Figure 1 and Figure 2).

### Pooled Prevalence of Obesity at T2D Diagnosis

**Figure 3.**
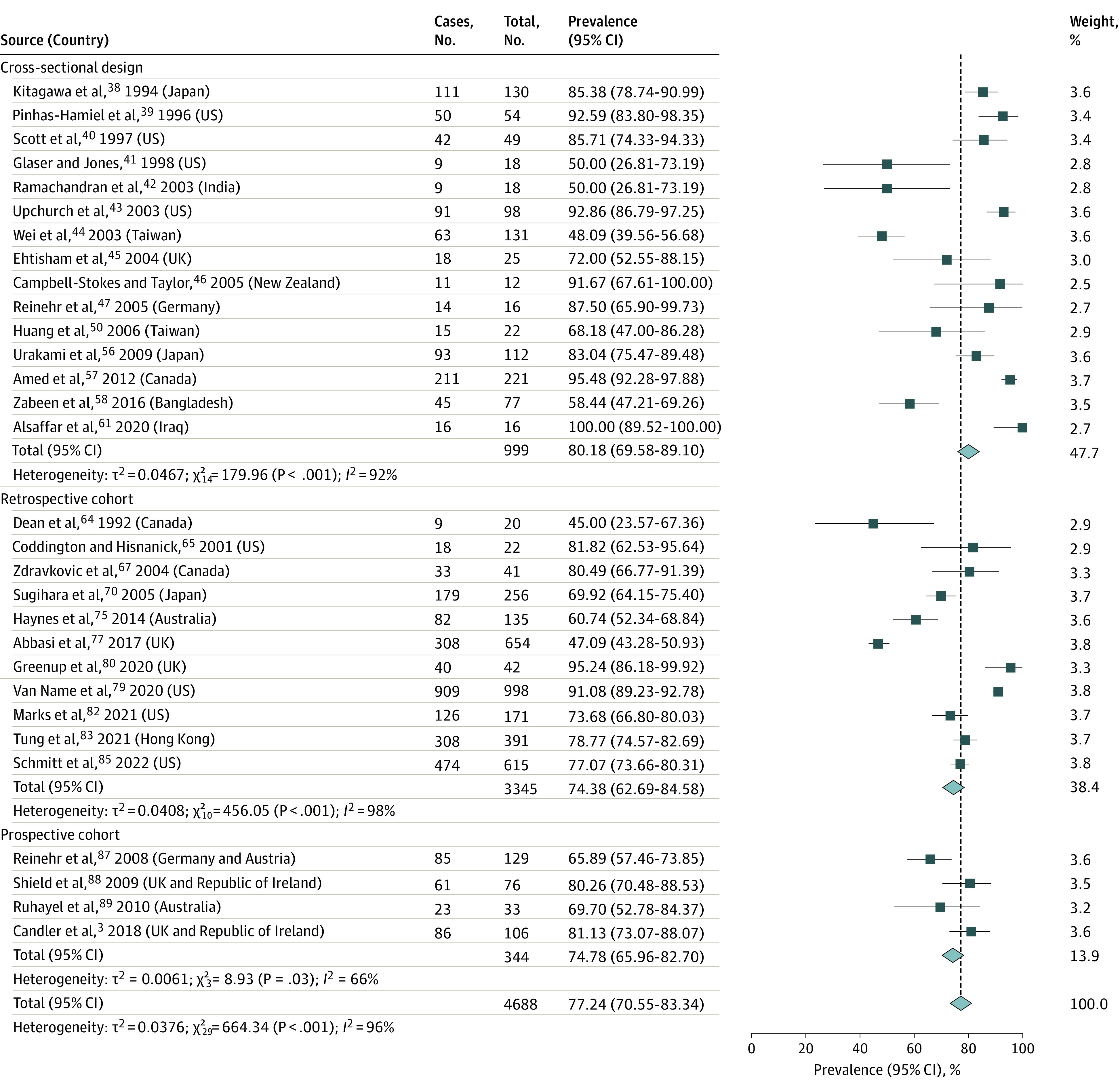
Pooled Obesity Prevalence at Pediatric Type 2 Diabetes Diagnosis Across All Included Studies, by Study Design

Data from 30 studies with 4688 participants reported an obesity prevalence in T2D at diagnosis of 77.24% (95% CI, 70.55%-83.34%; *I*^2^ = 96%; *P* < .001) (Figure 3).^3,38,39,40,41,42,43,44,45,46,47,50,56,57,58,61,64,65,67,70,75,77,79,80,82,83,85,87,88,89^ The pooled prevalence in cross-sectional studies was 80.18% (95% CI, 69.58%-89.10%; *I*^2^ = 92%; *P* < .001; n = 999)^38,39,40,41,42,43,44,45,46,47,50,56,57,58,61^ and 74.38% (95% CI, 62.69%-84.58%; *I*^2^ = 98%; *P* < .001; n = 3345) for retrospective cohort studies.^3,64,65,67,70,75,77,79,80,82,83,85,87,88,89^ The meta-analysis of prospective cohort studies found a prevalence of 74.78% (95% CI, 65.96%-82.70%; *I*^2^ = 66%; *P* = .03; n = 344).^3,87,88,89^

As these data indicated that some patients with T2D did not have obesity, we further characterized this population. There were wide variations in the prevalence of overweight and reference-range weight in the included studies (eTable 8 in Supplement 1). The prevalence of overweight ranged from 0.0% to 43.40% and normal weight from 0.0% to 43.60%.^3,11,40,42,43,45,48,49,50,51,52,53,54,58,60,63,69,71,72,74,75,77,78,79,81,83,84,87,88,89,92^

When assessing glycemic control and lipid homeostasis, studies reported a broad range of HbA_1c_ levels (4.5%-12.6% [to convert to proportion of total hemoglobin, multiply by 0.01]). Metaregression analysis revealed no significant correlations between obesity prevalence and mean HbA_1c_ levels (eTable 8 in Supplement 1).

The prevalence of dyslipidemia was 4.0% to 87.5% across 31 studies, with a mixed dyslipidemia profile including hypertriglyceridemia, high LDL cholesterol levels, and low HDL cholesterol levels. The metaregression analysis indicated significant associations between obesity and low HDL cholesterol levels (*P* = .04), but not hypercholesterolemia, hypertriglyceridemia, or elevated LDL cholesterol levels.

### Subgroup Analyses by Sex and Race

**Figure 4.**
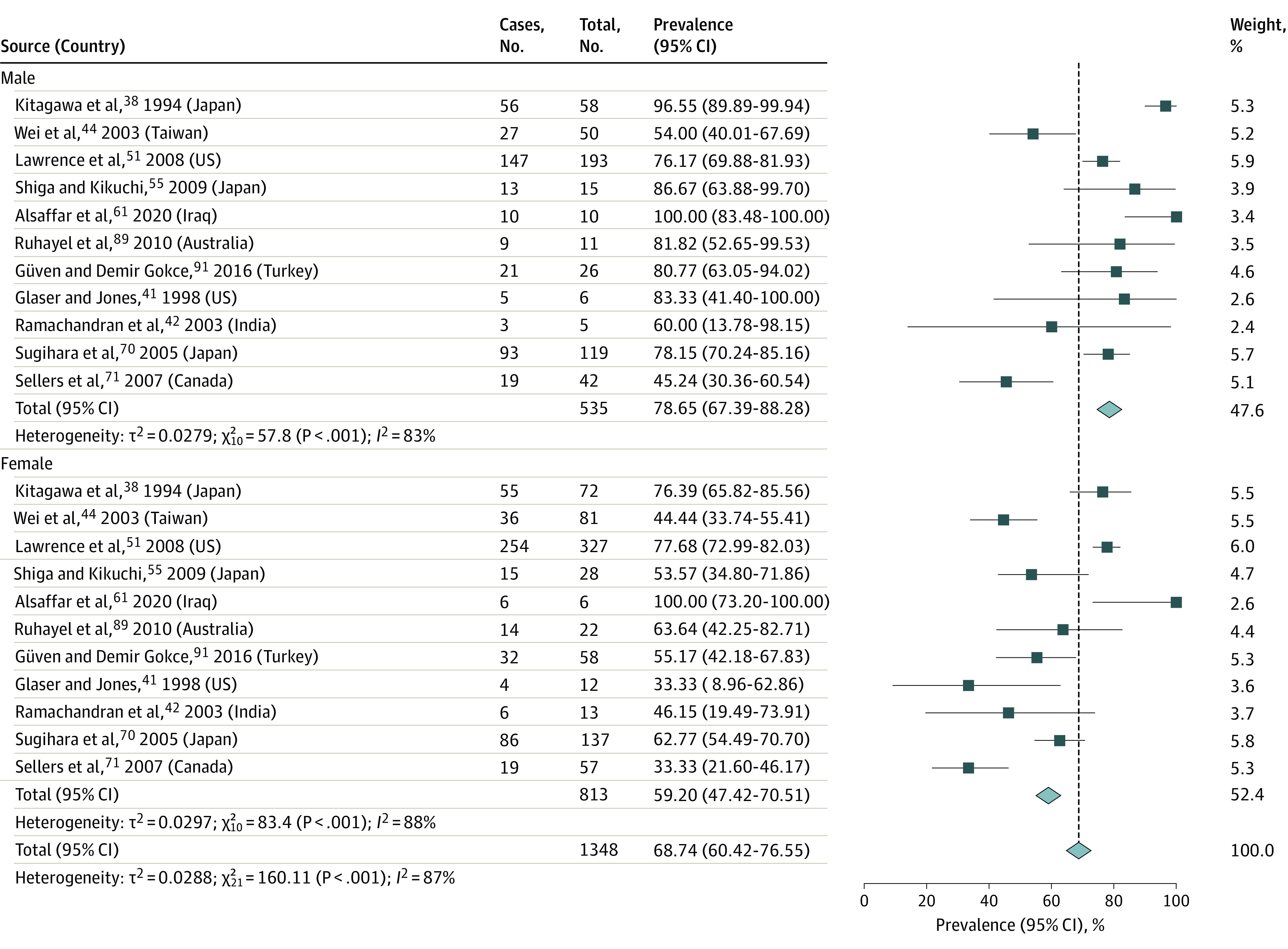
Prevalence of Obesity in Pediatric Type 2 Diabetes by Sex

The pooled prevalence of obesity in male participants with T2D was 78.65% (95% CI, 67.39%-88.28%; *I*^2^ = 83%; *P* < .001, n = 535), and the estimate was lower in female participants, at 59.20% (95% CI, 47.42%-70.51%; *I*^2^ = 88%; *P* < .001; n = 813) (Figure 4). The pooled OR of obesity prevalence for male vs female participants was 2.10 (95% CI, 1.33-3.31; *I*^2^ = 52%; *P* = .03) (eFigure 2 in Supplement 1).^38,41,42,44,51,55,61,70,71,89,91^

**Figure 5.**
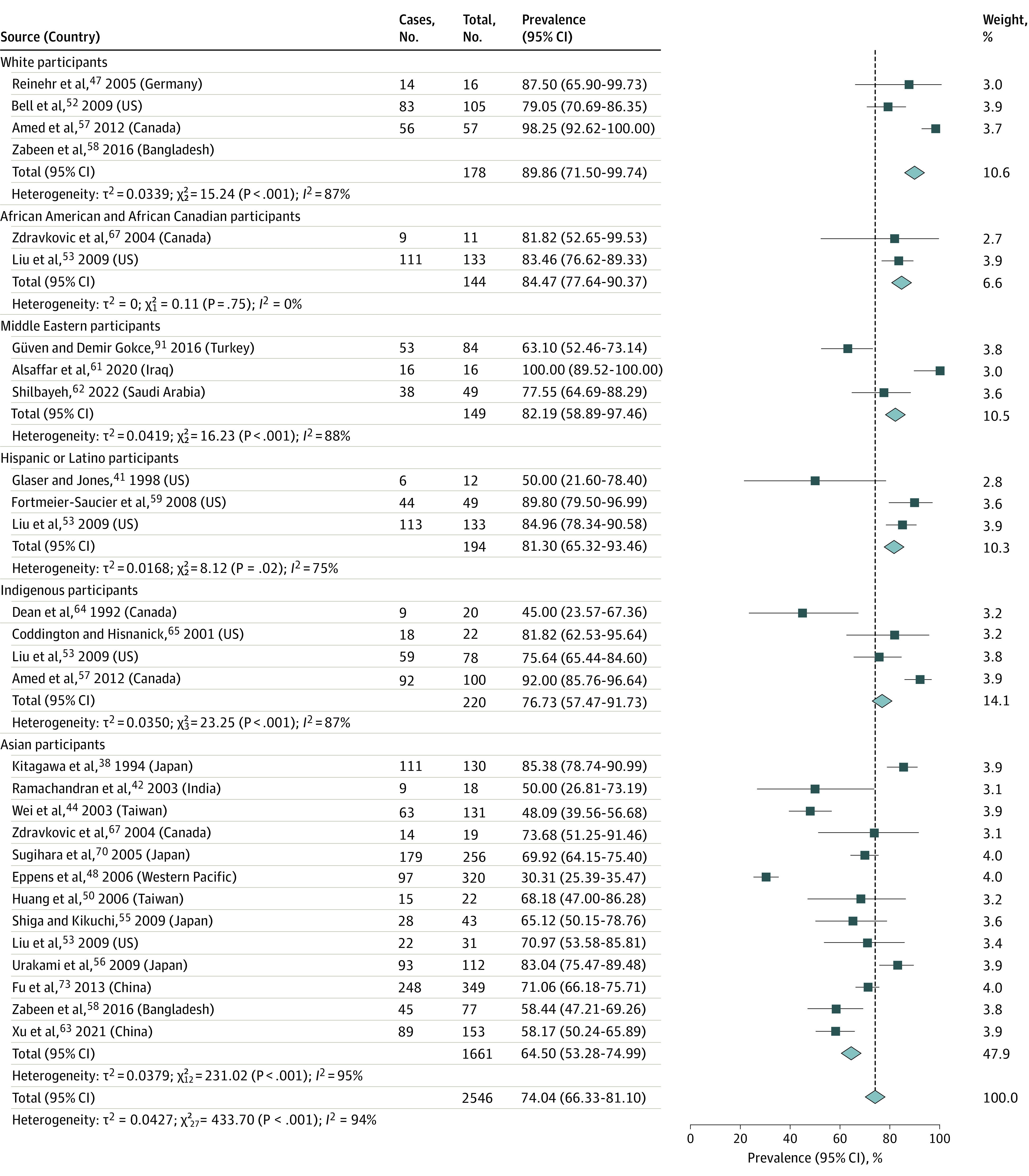
Prevalence of Obesity in Pediatric Type 2 Diabetes by Race

For race-reported data, the pooled obesity prevalence was 89.86% (95% CI, 71.50%-99.74%; *I*^2^ = 87%; *P* < .001; n = 178) in White patients,^47,52,57^ 84.47% (95% CI, 77.64%-90.37%; *I*^2^ = 0%; *P* = .75; n = 144) in African American and African Canadian patients,^53,67^ 82.19% (95% CI, 58.89%-97.46%; *I*^2^ = 88%; *P* < .001; n = 149) in Middle Eastern patients,^61,62,91^ 81.30% (95% CI, 65.32%-93.46%; *I*^2^ = 75%; *P* = .02; n = 194) in Hispanic and Latino patients,^41,53,59^ 76.73% (95% CI, 57.47%-91.73%; *I*^2^ = 87%; *P* < .001; n = 220) in Indigenous patients,^53,57,64,65^ and 64.50% (95% CI, 53.28%-74.99%; *I*^2^ = 95%; *P* < .001; n = 1661) in Asian patients (Figure 5).^38,42,44,48,50,54,55,56,58,63,67,70,73^ One study reported the prevalence of obesity at 72.20% in a mixed population of Asian and Pacific Islander patients (11 total patients),^54^ and another reported a prevalence of 64.70% in 51 Australian Indigenous patients and 93.50% in 49 Māori youths.^60^

Region-based analysis revealed that North America had the highest prevalence of obesity in patients with T2D at 81.14% (95% CI, 75.99%-85.83%; *I*^2^ = 94%; *P* < .001; n = 4779).^39,40,41,43,49,51,57,59,64,65,67,69,71,79,80,81,82,84,85,90,92^ The Middle East had the second highest, at 78.41% (95% CI, 68.29%-87.12%; *I*^2^ = 77%; *P* < .001; n = 547),^61,62,74,86,91^ followed by Oceania at 74.03% (95% CI, 55.52%-89.15%; *I*^2^ = 96%; *P* < .001; n = 871),^11,46,48,60,68,75,76,89^ Asia at 68.54% (95% CI, 61.16%-75.51%; *I*^2^ = 89%; *P* < .001; n = 1682),^4,38,42,50,55,56,58,63,70,73,83^ and Europe at 68.30% (95% CI, 54.84%-80.43%; *I*^2^ = 92%, *P* < .001, n = 1063) (eFigure 3 in Supplement 1).^3,45,47,72,77,78,87,88^

We also analyzed the prevalence of reference-range BMI measures in patients with T2D by region. The highest prevalence was in studies from Oceania (16.43%; 95% CI, 5.37%-31.51%; *I*^2^ = 95%; *P* < .001; n = 836)^11,48,60,68,75,89^ and Asia (13.95%; 95% CI, 4.52%-26.93%; *I*^2^ = 91%; *P* < .001; n = 661),^42,50,58,63,83^ whereas Europe (9.52%; 95% CI, 0.46%-25.83%; *I*^2^ = 97%; *P* < .001; n = 1063),^3,45,47,72,77,78,87,88^ the Americas (4.21%; 95% CI, 1.55%-7.93%; *I*^2^ = 95%; *P* < .001; n = 3568)^40,43,49,51,69,71,79,80,81,84,90,92^ and the Middle East (1.26%; 95% CI, 0.00%-7.32%; *I*^2^ = 0%; *P* < .001; n = 54)^61,74^ had lower prevalence (eFigure 4 in Supplement 1).

### Sensitivity Analyses

The sensitivity analyses assessed whether the diagnostic criteria for obesity and diabetes, autoimmunity, or the potential for the initial weight loss at diabetes presentation would affect obesity prevalence. There were no studies with a high risk of bias.

Most studies used the 95th percentile of BMI for age and sex to define obesity.^26^ However, some studies used the adult obesity cutoff (BMI [calculated as weight in kilograms divided by height in meters squared] ≥30), and some did not report the obesity definition used. These studies were removed in the sensitivity analysis (eTable 9 in Supplement 1).^38,39,41,42,47,49,55,56,57,61,70,72,75,78,83,85,91^ Three studies enrolled only patients with overweight or obesity.^47,79,90^ We conducted another sensitivity analysis for prevalence estimates excluding these studies. The overall pooled prevalence differed very slightly, with substantial heterogeneity noted.

Another sensitivity analysis excluded studies with uncertain or unspecified T2D diagnostic criteria.^43,49,51,56,59,64,69,70,72,75,76,78,84,91^ We also performed sensitivity analyses excluding patients with positive tests for islet cell, glutamic acid decarboxylase, and islet tyrosine phosphatase 2 pancreatic autoantibodies (n = 55).^43,45,46,64,80,91^ The results of these analyses led to a pooled prevalence of obesity in the pediatric T2D population of 74.81% (95% CI, 69.72%-79.59%; *I*^2^ = 96%; *P* < .001) (eTable 9 in Supplement 1). We also excluded studies of patients who presented with weight loss.^3,39,40,43,49,65,67,72,74,80,82,83,84,86^ The pooled obesity prevalence was 72.87% (95% CI, 66.58%-78.75%, *I*^2^ = 97%, *P* < .001). We also performed a sensitivity analysis removing studies that specifically excluded patients with MODY based on genetic testing results,^3,39,45,51,60,72,80,82,86,87,88^ and the pooled prevalence was 78.87%; (95% CI, 74.70%-82.77%; *I*^2^ = 85%; *P* < .001). In conclusion, our results did not differ significantly with any of these sensitivity analyses.

### Publication Bias

No publication bias was identified for the prevalence of obesity at study visit or diagnosis from the funnel plots or Egger tests. eFigures 5 and 6 in Supplement 1 present these analyses.

### Risk of Bias Within Studies

Studies had either a low (n = 32)^3,38,39,44,45,46,48,50,51,52,53,58,60,61,62,63,64,65,67,70,71,73,74,77,81,83,84,85,86,88,90,92^ or moderate risk of bias (n = 25)^11,40,41,42,43,47,49,54,55,56,57,59,66,68,69,72,75,76,78,79,80,82,87,89,91^ (eTable 10 in Supplement 1). Risk of bias was present in studies with sampling frames that were not a close representation of the target population, likely driven by the rarity of the diagnosis of T2D in children^38,40,42,43,48,49,55,56,57,59,66,69,72,78,80,91^ or used convenience sampling instead of a census or random sample selection.^40,43,49,55,56,57,59,66,78,79,80,90,91^

Some studies had 25% or higher rates of missing data, potentially indicating a nonresponse bias.^11,43,47,54,60,62,63,66,69,70,75,76,82,87,89^ In some studies, it was unclear that all individuals were examined using the same methods, as participants were tested in different clinics with no reported standardized protocols.^3,41,46,57,68,69,77,79,80,87,88^ Most studies only assessed obesity in patients in a particular city or clinic.^11,39,40,41,42,43,47,49,50,52,54,55,56,58,59,61,64,65,66,67,68,71,72,74,75,76,78,80,81,82,84,85,89,91^

### Level of Evidence

Based on Oxford Centre for Evidence-Based Medicine criteria, 28 studies (49.1%) had a level of evidence of 1,^3,11,38,39,44,48,51,52,53,54,58,60,63,69,70,71,73,75,77,81,82,83,84,85,86,87,88,92^ 16 (28.1%) had a level of evidence of 2,^41,42,45,46,47,50,61,62,64,65,67,68,72,74,76,89^ and 13 (22.8%) had a level of evidence of 3.^40,43,49,55,56,57,59,66,78,79,80,90,91^ A significant portion of studies did not use a random sample or census to estimate prevalence, which may limit the assessment of the level of evidence of the prevalence estimate.

## Discussion

Childhood obesity is a global health crisis affecting approximately 340 million children and is a major driver of T2D risk.^93,94,95^ Understanding the contribution of body mass to the evolution of insulin resistance, glucose intolerance, and T2D and its comorbidities and complications is crucial for creating personalized interventions to improve outcomes.

While acknowledging the low to moderate risk of bias, variable levels of evidence, and high heterogeneity, up to 1 in 4 children with T2D do not have obesity, and some have reference-range body mass measures. While the obvious conclusions of the analysis are that there are limitations of BMI-based measures to predict diabetes and that mechanisms beyond obesity are involved in T2D evolution in children, the selection for screening of at-risk children to establish the diagnosis becomes more complex. Guidelines generally look for elevated body mass measures as a main screening indication. While factors such as ethnicity and in utero exposure to diabetes are already combined with BMI-based measures to justify screening, more sophisticated prediabetes and diabetes prediction models are needed to justify a broader screening approach. These models may need to incorporate family history, in utero exposure to diabesity, lifestyle factors, hormones, puberty, growth, sex, race and ethnicity, markers of insulin resistance, insulin production capacity, and others to refine the identification of those in need of screening.^21,22,23^

The 2 main mechanisms driving T2D include insulin resistance and insulin deficiency. In children with T2D, beta cell dysfunction manifests with substantial impairments in first- and second-phase insulin secretion,^96,97,98^ and children with T2D and normal weight have lower insulin secretory capacity than patients with T2D and obesity.^99^ The decline in beta cell function in children with T2D is 20% or greater per annum, which is almost double the rate seen in adult T2D.^97,100,101,102,103,104^ Patients with positive autoimmunity have more severe insulin deficiency compared with patients with autoantibody-negative T2D, who are more likely to have severe insulin resistance.^105^ The pathogenic mechanisms driving diabetes in these 2 subgroups may be different and need further study.

Recent evidence from adult studies suggests that adult diabetes subtypes can be classified based on age, BMI, diabetes-related autoantibodies, HbA_1c_, islet function, and insulin resistance.^106^ This classification system defined several patient subpopulations, including those with normal BMI with insulin deficiency with or without islet autoimmunity, those with high BMI with or without severe insulin resistance, and a mild form of diabetes of old age.^106^ Similarly, there may be subtypes of pediatric T2D in which children may or may not have obesity and autoimmunity, with varying degrees of metabolic end-organ insulin resistance or defects in beta cell insulin secretion. These phenotypes may be driven by glucolipotoxicity, genetic defects of beta cells, epigenetics, autoimmunity, and inflammation as drivers of diabetes risk. Further studies are needed to define the different potential subgroups of children with T2D.

While it is already known that more girls develop T2D than boys,^1,2,3,4,5^ our data suggest that boys with T2D were more likely to have obesity than girls. The mechanisms driving sex differences in T2D risk in children are not fully understood.^107^ Increased adiposity and insulin resistance are physiological changes during puberty, and increased weight during puberty may be driven by and contribute to hyperinsulinemia.^107^ However, obesity is likely one factor that augments peripubertal insulin resistance and may contribute to diabetes risk.^107^

Although patients of other racial and ethnic groups are at a higher risk of T2D than White patients,^2,108^ there were only a few studies that reported on the prevalence of obesity in different subpopulations, with some overlap of confidence intervals. Within this limitation, Asian children with T2D tended to have a lower prevalence of obesity than the other racial groups; there is evidence that these children develop T2D at lower BMI levels than other groups.^18^ There are subgroups of children in Japan with a nonobese, nonautoimmune phenotype with T2D and reduced insulin secretion with insulin resistance, and female patients with a history of low birth weight are at particular risk.^109^ Having a higher total and visceral adiposity than other groups are postulated mechanisms driving T2D in this population.^19^

While African American and Hispanic and Latino children have higher rates of T2D than White children, these populations have similar rates of obesity with T2D. Further analysis is needed to understand the mechanisms driving racial and ethnic variations in T2D risk.

The identification of patients with T2D and normal body mass defines a path to T2D genesis in which obesity is not a factor. It is likely that obesity-independent insulin secretory defects and insulin resistance and other factors play important roles in the development of diabetes in this group, and further analyses of this group are essential.

A trend that emerged from the analysis was that most studies reported a mean or median HbA_1c_ level greater than 7.0%, which is higher than targeted glycemic control.^21,22^ These results confirm the challenges in achieving adequate glycemic control in this population, the attainment of which can reduce diabetes-related complications such as retinopathy and albuminuria.^110,111^

We did not identify significant associations between the prevalence of obesity and dyslipidemia or HbA_1c_ levels in patients with pediatric T2D. As not all studies reported data on HbA_1c_ levels and dyslipidemia, it is possible that there was insufficient power to detect a significant association or that obesity-driven insulin resistance has an indirect association with dyslipidemia. Insulin resistance disrupts hepatic fatty acids flux, reduces muscle fatty acid uptake, and upregulates adipose tissue lipolysis due to resistance to the antilipolytic effects of insulin that can propagate dyslipidemia.^112^

### Limitations

This study has limitations. One limitation of the study is the high heterogeneity and that not all studies reported on the exclusion of MODY or other forms of diabetes. The high heterogeneity encountered affects the certainty of our estimate. While subgroup analysis by racial and ethnic groups did identify different prevalence values for different races, this analysis did not fully explain the heterogeneity, and thus, factors beyond race and ethnicity likely affect the association of obesity and T2D. Patients with MODY tend to have lower BMI than those with T2D,^113^ so if patients had MODY, that could lower the obesity prevalence estimate. However, it is unlikely that a large enough proportion of patients had MODY to affect our results, given that MODY is rare, its diagnosis requires the fulfillment of certain diagnostic criteria, and there are practical and cost considerations that limit having large screening programs for MODY in the T2D population.^21^ The sensitivity analyses demonstrated similar prevalence of obesity in T2D when studies with no genetic testing for MODY were removed (eTable 9 in Supplement 1), so it is unlikely that this issue has significant implications on the results.

Importantly, as clinical guidelines generally use overweight and obesity as one of the main criteria to screen for T2D in children,^21,22,23^ it is possible that the prevalence value is underestimated due to the likelihood that children with normal body mass are not necessarily screened for T2D. However, population-based screening is not cost-effective in most parts of the world, and clarification of the screening criteria is warranted to include those with normal body mass. In addition, the expansion of the visceral adipose compartment is a crucial risk factor for developing T2D independent of BMI and total adiposity.^114,115,116^ There were no data on visceral adiposity in the included studies, and this possibility requires further study.

## Conclusions

In this study, while obesity was an important risk factor for the development of T2D in children, not all patients with T2D had obesity. Screening for and diagnosing T2D may consider obesity as a risk factor for T2D but not a prerequisite to screening when other risk factors are present.

Understanding the causes of T2D in children without obesity is crucial to define the etiology of their diabetes and to create effective management strategies for this cohort. Further research is needed to evaluate the causes of sex- and race and ethnicity–based associations of diabetes with obesity and explore additional factors that may affect the risk of developing T2D apart from obesity in children.

## Notes

Supplement 1. **eTable 1. **Search Strategy: MEDLINE **eTable 2. **Search Strategy: Embase **eTable 3. **Search Strategy: CINAHL **eTable 4. **Search Strategy: Cochrane Library: Cochrane Central Register of Controlled Trials and Cochrane Database of Systematic Reviews **eTable 5. **Search Strategy: Web of Science: Conference Proceedings Citation Index-Science **eTable 6. **Characteristics of Included Studies **eTable 7. **Symptoms at Presentation and Risk Factors of Patients in Included and Type 2 Diabetes Diagnostic Criteria Used Across Studies **eTable 8. **Prevalence of Type 2 Diabetes in Patients Without Obesity and Association With Glycemic Control and Dyslipidemia **eTable 9. **Results of Sensitivity Analyses **eTable 10. **Risk of Bias of Included Studies **eFigure 1. **Study Flow Diagram **eFigure 2. **Forest Plot Illustrating Odds Ratio of Obesity in Pediatric T2DM by Sex **eFigure 3. **Forest Plot Illustrating Prevalence of Obesity in Pediatric T2DM by Region **eFigure 4. **Forest Plot Illustrating Prevalence of Normal BMI-Based Measures in Pediatric T2DM by Region **eFigure 5. **Funnel Plot for Publication Bias for Pooled Prevalence of Obesity in Pediatric Type 2 Diabetes **eFigure 6. **Funnel Plot for Publication Bias for Pooled Prevalence of Obesity at Type 2 Diabetes Diagnosis in Patients With Pediatric Type 2 Diabetes
eReferences.

Supplement 2.
Data Sharing Statement
