# Supplementary material for: The Prevalence of Obesity Among Children With Type 2 Diabetes: A Systematic Review and Meta-analysis
Source: JAMA Netw Open. 2022 Dec 15;5(12):e2247186. doi: 10.1001/jamanetworkopen.2022.47186 (PMC9856349; doi:10.1001/jamanetworkopen.2022.47186)
Supplement: Supplement 1. — eTable 1. Search Strategy: MEDLINE eTable 2. Search Strategy: Embase eTable 3. Search Strategy: CINAHL eTable 4. Search Strategy: Cochrane Library: Cochrane Central Register of Controlled Trials and Cochrane Database of Systematic Reviews eTable 5. Search Strategy: Web of Science: Conference Proceedings Citation Index-Science eTable 6. Characteristics of Included Studies eTable 7. Symptoms at Presentation and Risk Factors of Patients in Included and Type 2 Diabetes Diagnostic Criteria Used Across Studies eTable 8. Prevalence of Type 2 Diabetes in Patients Without Obesity and Association With Glycemic Control and Dyslipidemia eTable 9. Results of Sensitivity Analyses eTable 10. Risk of Bias of Included Studies eFigure 1. Study Flow Diagram eFigure 2. Forest Plot Illustrating Odds Ratio of Obesity in Pediatric T2DM by Sex eFigure 3. Forest Plot Illustrating Prevalence of Obesity in Pediatric T2DM by Region eFigure 4. Forest Plot Illustrating Prevalence of Normal BMI-Based Measures in Pediatric T2DM by Region eFigure 5. Funnel Plot for Publication Bias for Pooled Prevalence of Obesity in Pediatric Type 2 Diabetes eFigure 6. Funnel Plot for Publication Bias for Pooled Prevalence of Obesity at Type 2 Diabetes Diagnosis in Patients With Pediatric Type 2 Diabetes eReferences. [file jamanetwopen-e2247186-s001.pdf]

Supplemental Online Content

Cioana M, Deng J, Nadarajah A, et al. The prevalence of obesity among children with type 2 diabetes: a systematic review and meta-analysis. *JAMA Netw Open.* 2022;5(12):e2247186. doi:10.1001/jamanetworkopen.2022.47186

eTable 1. Search Strategy: MEDLINE

eTable 2. Search Strategy: Embase

eTable 3. Search Strategy: CINAHL

eTable 4. Search Strategy: Cochrane Library: Cochrane Central Register of Controlled Trials and Cochrane Database of Systematic Reviews

eTable 5. Search Strategy: Web of Science: Conference Proceedings Citation Index-Science

eTable 6. Characteristics of Included Studies

eTable 7. Symptoms at Presentation and Risk Factors of Patients in Included and Type 2 Diabetes Diagnostic Criteria Used Across Studies

eTable 8. Prevalence of Type 2 Diabetes in Patients Without Obesity and Association With Glycemic Control and Dyslipidemia

eTable 9. Results of Sensitivity Analyses

eTable 10. Risk of Bias of Included Studies

eFigure 1. Study Flow Diagram

eFigure 2. Forest Plot Illustrating Odds Ratio of Obesity in Pediatric T2DM by Sex

eFigure 3. Forest Plot Illustrating Prevalence of Obesity in Pediatric T2DM by Region

eFigure 4. Forest Plot Illustrating Prevalence of Normal BMI-Based Measures in Pediatric T2DM by Region

eFigure 5. Funnel Plot for Publication Bias for Pooled Prevalence of Obesity in Pediatric Type 2 Diabetes

eFigure 6. Funnel Plot for Publication Bias for Pooled Prevalence of Obesity at Type 2 Diabetes Diagnosis in Patients With Pediatric Type 2 Diabetes

eReferences.

This supplemental material has been provided by the authors to give readers additional information about their work.

© 2022 Cioana M et al. *JAMA Network Open.*

eTable 1. Search Strategy: MEDLINE

1	exp Diabetes Mellitus, Type 2/
2	NIDDM.ti,ab,kf.
3	MODY.ti,ab,kf.
4	t2d*.ti,ab,kf.
5	((typ* two or typ?two or typ* 2 or typ* II or typ?2 or typ?II or typ* ii or typ?ii) adj4 diabet*).ti,ab,kf.
6	((non insulin or noninsulin or late or adult* or matur* or slow or stabl*) adj4 diabet*).ti,ab,kf.
7	((ketoresist* or keto* resist* or keto* prone) adj4 diabet*).ti,ab,kf.
8	or/1-7
9	exp Child/
10	child*.ti,ab,kf.
11	adolescen*.ti,ab,kf.
12	exp Adolescent/
13	youth*.ti,ab,kf.
14	teenage*.ti,ab,kf.
15	preadolescen*.ti,ab,kf.
16	Pediatrics/
17	p?ediatric*.ti,ab,kf.
18	pe?diatric*.ti,ab,kf.
19	or/9-18
20	8 and 19
21	exp body weight changes/
22	exp Obesity/
23	(obese or obesit*).ti,ab,kf.
24	(weight* adj2 (gain* or control* or manage* or maintain* or maintenance or increas* or cycl* or fluctuation* or variation*)).ti,ab,kf.
25	Overweight/
26	(overweight* or over weight*).ti,ab,kf.
27	exp Body Fat Distribution/
28	adipos*.ti,ab,kf.
29	Skinfold Thickness/
30	"body fat distribut*".ti,ab,kf.
31	or/21-30
32	20 and 31
33	32 not (animals/ not (humans/ and animals/))
34	Prevalence/
35	prevalence.ti,ab,kf.
36	prevalence studies/

© 2022 Cioana M et al. *JAMA Network Open.*

37	Incidence/
38	incidence studies/
39	incidence.ti,ab,kf.
40	Epidemiology/
41	epidemiolog*.ti,ab,kf.
42	ep.fs.
43	epidemiologic methods/ or epidemiological monitoring/ or sentinel surveillance/
44	exp epidemiologic studies/
45	case-control.ti,ab,kf.
46	cohort.ti,ab,kf.
47	prospective.ti,ab,kf.
48	longitudinal.ti,ab,kf.
49	retrospective.ti,ab,kf.
50	cross sectional.ti,ab,kf.
51	correlational.ti,ab,kf.
52	or/34-51
53	33 and 52
54	remove duplicates from 53

© 2022 Cioana M et al. *JAMA Network Open.*

eTable 2. Search Strategy: Embase

1	non insulin dependent diabetes mellitus/
2	NIDDM.ti,ab,kw.
3	MODY.ti,ab,kw.
4	t2d*.ti,ab,kw.
5	((typ* two or typ?two or typ* 2 or typ* II or typ?2 or typ?II or typ* ii or typ?ii) adj4 diabet*).ti,ab,kw.
6	((non insulin or noninsulin or late or adult* or matur* or slow or stabl*) adj4 diabet*).ti,ab,kw.
7	((ketoresist* or keto* resist*) adj6 diabet*).ti,ab,kw.
8	or/1-7
9	exp child/
10	child*.ti,ab,kw.
11	adolescent/
12	adolescen*.ti,ab,kw.
13	youth*.ti,ab,kw.
14	teenage*.ti,ab,kw.
15	preadolescen*.ti,ab,kw.
16	pediatrics/
17	p?ediatric*.ti,ab,kw.
18	pe?diatric*.ti,ab,kw.
19	or/9-18
20	8 and 19
21	weight change/
22	exp obesity/
23	(obese or obesit*).ti,ab,kw.
24	(weight* adj2 (gain* or control* or manage* or maintain* or maintenance or increas* or cycl* or fluctuation* or variation* or excess*)).ti,ab,kw.
25	(overweight* or over weight*).ti,ab,kw.
26	exp adipose tissue/
27	adipos*.ti,ab,kw.
28	"body fat distribut*".ti,ab,kw.
29	or/21-28
30	20 and 29
31	prevalence/
32	prevalence.ti,ab,kw.
33	incidence/
34	incidence.ti,ab,kw.
35	epidemiology/
36	epidemiolog*.ti,ab,kw.

37	ep.fs.
38	epidemiological monitoring/
39	sentinel surveillance/
40	case-control.ti,ab,kw.
41	cohort.ti,ab,kw.
42	prospective.ti,ab,kw.
43	longitudinal.ti,ab,kw.
44	retrospective.ti,ab,kw.
45	cross sectional.ti,ab,kw.
46	correlational.ti,ab,kw.
47	or/31-46
48	30 and 47
49	48 not (animals/ not (humans/ and animals/))
50	remove duplicates from 49

© 2022 Cioana M et al. *JAMA Network Open.*

eTable 3. Search Strategy: CINAHL

#	Query	Limiters/Expanders	Last Run Via
S1	(MH "Child+")	Search modes - Boolean/Phrase	Interface - EBSCOhost Research Databases<br>Search Screen - Advanced Search<br>Database - CINAHL
S2	"child*"	Search modes - Boolean/Phrase	Interface - EBSCOhost Research Databases<br>Search Screen - Advanced Search<br>Database - CINAHL
S3	(MH "Adolescence+")	Search modes - Boolean/Phrase	Interface - EBSCOhost Research Databases<br>Search Screen - Advanced Search<br>Database - CINAHL
S4	"youth*"	Search modes - Boolean/Phrase	Interface - EBSCOhost Research Databases<br>Search Screen - Advanced Search<br>Database - CINAHL
S5	"teenage*"	Search modes - Boolean/Phrase	Interface - EBSCOhost Research Databases<br>Search Screen - Advanced Search<br>Database - CINAHL
S6	(MH "Pediatrics")	Search modes - Boolean/Phrase	Interface - EBSCOhost Research Databases<br>Search Screen - Advanced Search<br>Database - CINAHL
S7	"p?ediatric*"	Search modes - Boolean/Phrase	Interface - EBSCOhost Research Databases<br>Search Screen - Advanced Search<br>Database - CINAHL
S8	"p#ediatric*"	Search modes - Boolean/Phrase	Interface - EBSCOhost Research Databases<br>Search Screen - Advanced Search<br>Database - CINAHL
S9	"pe#diatric*"	Search modes - Boolean/Phrase	Interface - EBSCOhost Research Databases<br>Search Screen - Advanced Search<br>Database - CINAHL
S10	"pediatric*"	Search modes - Boolean/Phrase	Interface - EBSCOhost Research Databases<br>Search Screen - Advanced Search<br>Database - CINAHL
S11	"preadolescen*"	Search modes - Boolean/Phrase	Interface - EBSCOhost Research Databases<br>Search Screen - Advanced Search<br>Database - CINAHL

© 2022 Cioana M et al. *JAMA Network Open.*

S12	S1 OR S2 OR S3 OR S4 OR S5 OR S6 OR S7 OR S8 OR S9 OR S10 OR S11	Search modes - Boolean/Phrase	Interface - EBSCOhost Research Databases<br>Search Screen - Advanced Search<br>Database - CINAHL
S13	(MH "Diabetes Mellitus, Type 2")	Search modes - Boolean/Phrase	Interface - EBSCOhost Research Databases<br>Search Screen - Advanced Search<br>Database - CINAHL
S14	"NIDDM"	Search modes - Boolean/Phrase	Interface - EBSCOhost Research Databases<br>Search Screen - Advanced Search<br>Database - CINAHL
S15	"MODY"	Search modes - Boolean/Phrase	Interface - EBSCOhost Research Databases<br>Search Screen - Advanced Search<br>Database - CINAHL
S16	"T2D*"	Search modes - Boolean/Phrase	Interface - EBSCOhost Research Databases<br>Search Screen - Advanced Search<br>Database - CINAHL
S17	(typ* two or typ?two or typ* 2 or typ* II or typ?2 or typ? II) N4 diabet*	Search modes - Boolean/Phrase	Interface - EBSCOhost Research Databases<br>Search Screen - Advanced Search<br>Database - CINAHL
S18	(non insulin or noninsulin or late or adult* or matur* or slow or stabl*) N4 diabet*	Search modes - Boolean/Phrase	Interface - EBSCOhost Research Databases<br>Search Screen - Advanced Search<br>Database - CINAHL
S19	(ketoresist* or keto* resist* or keto* prone) adj4 diabet*	Search modes - Boolean/Phrase	Interface - EBSCOhost Research Databases<br>Search Screen - Advanced Search<br>Database - CINAHL
S20	S13 OR S14 OR S15 OR S16 OR S17 OR S18 OR S19	Search modes - Boolean/Phrase	Interface - EBSCOhost Research Databases<br>Search Screen - Advanced Search<br>Database - CINAHL
S21	S12 AND S20	Search modes - Boolean/Phrase	Interface - EBSCOhost Research Databases<br>Search Screen - Advanced Search<br>Database - CINAHL
S22	(MH "Obesity+")	Search modes - Boolean/Phrase	Interface - EBSCOhost Research Databases<br>Search Screen - Advanced Search<br>Database - CINAHL
S23	(MH "Body Weight Changes")	Search modes - Boolean/Phrase	Interface - EBSCOhost Research Databases

© 2022 Cioana M et al. *JAMA Network Open.*

			Search Screen - Advanced Search<br>Database - CINAHL
S24	weight* N2 (chang* or gain* or control* or manage* or maintain* or increas* or cycl* or fluctuation* or variation*)	Search modes - Boolean/Phrase	Interface - EBSCOhost Research Databases<br>Search Screen - Advanced Search<br>Database - CINAHL
S25	"overweight" or "over weight"	Search modes - Boolean/Phrase	Interface - EBSCOhost Research Databases<br>Search Screen - Advanced Search<br>Database - CINAHL
S26	(MH "Adipose Tissue Distribution")	Search modes - Boolean/Phrase	Interface - EBSCOhost Research Databases<br>Search Screen - Advanced Search<br>Database - CINAHL
S27	"adipos*" or "body fat distribution*"	Search modes - Boolean/Phrase	Interface - EBSCOhost Research Databases<br>Search Screen - Advanced Search<br>Database - CINAHL
S28	S22 OR S23 OR S24 OR S25 OR S26 OR S27	Search modes - Boolean/Phrase	Interface - EBSCOhost Research Databases<br>Search Screen - Advanced Search<br>Database - CINAHL
S29	S21 AND S28	Search modes - Boolean/Phrase	Interface - EBSCOhost Research Databases<br>Search Screen - Advanced Search<br>Database - CINAHL
S30	(MH "Prevalence")	Search modes - Boolean/Phrase	Interface - EBSCOhost Research Databases<br>Search Screen - Advanced Search<br>Database - CINAHL
S31	"prevalence"	Search modes - Boolean/Phrase	Interface - EBSCOhost Research Databases<br>Search Screen - Advanced Search<br>Database - CINAHL
S32	(MH "Cross Sectional Studies")	Search modes - Boolean/Phrase	Interface - EBSCOhost Research Databases<br>Search Screen - Advanced Search<br>Database - CINAHL
S33	"cross section*"	Search modes - Boolean/Phrase	Interface - EBSCOhost Research Databases<br>Search Screen - Advanced Search<br>Database - CINAHL
S34	(MH "Incidence")	Search modes - Boolean/Phrase	Interface - EBSCOhost Research Databases

© 2022 Cioana M et al. *JAMA Network Open.*

			Search Screen - Advanced Search<br>Database - CINAHL
S35	"incidence"	Search modes - Boolean/Phrase	Interface - EBSCOhost Research Databases<br>Search Screen - Advanced Search<br>Database - CINAHL
S36	(MH "Epidemiology")	Search modes - Boolean/Phrase	Interface - EBSCOhost Research Databases<br>Search Screen - Advanced Search<br>Database - CINAHL
S37	"epidemiolog*"	Search modes - Boolean/Phrase	Interface - EBSCOhost Research Databases<br>Search Screen - Advanced Search<br>Database - CINAHL
S38	(MH "Epidemiological Research")	Search modes - Boolean/Phrase	Interface - EBSCOhost Research Databases<br>Search Screen - Advanced Search<br>Database - CINAHL
S39	(MH "Prospective Studies") OR (MH "Cross Sectional Studies") OR (MH "Case Control Studies") OR (MH "Correlational Studies")	Search modes - Boolean/Phrase	Interface - EBSCOhost Research Databases<br>Search Screen - Advanced Search<br>Database - CINAHL
S40	"case control" or "cohort" or "prospective" or "retrospective" or "longitudinal" or "correlational"	Search modes - Boolean/Phrase	Interface - EBSCOhost Research Databases<br>Search Screen - Advanced Search<br>Database - CINAHL
S41	S30 OR S31 OR S32 OR S33 OR S34 OR S35 OR S36 OR S37 OR S38 OR S39 OR S40	Search modes - Boolean/Phrase	Interface - EBSCOhost Research Databases<br>Search Screen - Advanced Search<br>Database - CINAHL
S42	(S29 AND S41) NOT (MH "Animals")	Search modes - Boolean/Phrase	Interface - EBSCOhost Research Databases<br>Search Screen - Advanced Search<br>Database - CINAHL

eTable 4. Search Strategy: Cochrane Library: Reviews and Trials

	Title Abstract Keyword	'child* OR youth* OR teenage* OR adolescen* OR pediatric* OR preadolescen* OR p?ediatric* OR pe?diatric*
AND	Title Abstract Keyword	NIDDM OR MODY OR t2d OR typ* two NEAR diabet* OR typ?two NEAR diabet* OR typ* 2 NEAR diabet* OR typ* II NEAR diabet* OR typ?2 NEAR diabet* OR typ?II NEAR diabet* OR typ* ii NEAR diabet* OR typ?ii NEAR diabet* OR non insulin NEAR diabet* OR noninsulin NEAR diabet* OR late or adult* NEAR diabet* OR matur* NEAR diabet* OR slow NEAR diabet* OR stabl* NEAR diabet* OR ketoresist* NEAR diabet* OR keto* resist* NEAR diabet* OR keto* prone NEAR diabet*
AND	Title Abstract Keyword	obese OR obesit* OR weight* NEAR(gain* or control* or manage* or maintain* or maintenance or increas* or cycl* or fluctuation* or variation*) or overweight* or over weight* or adipos* or "body fat distribut*"
(Word variations have been searched)		

© 2022 Cioana M et al. *JAMA Network Open.*

eTable 5. Search Strategy - Web of Science: Conference Proceedings Citation Index-Science (CPCI- S) - 1990-present

#1	TS=(child* OR youth* OR teenage* OR adolescen* OR pediatric* OR preadolescen* OR p?<br>ediatric* OR pe?diatric*)
#2	TI=(child* OR youth* OR teenage* OR adolescen* OR pediatric* OR preadolescen* OR p?<br>ediatric* OR pe?diatric*)
#3	#1 OR #2
#4	TS=(NIDDM OR MODY OR t2d OR typ* two NEAR/4 diabet* OR typ?two NEAR/4 diabet* OR typ* 2 NEAR/4 diabet* OR typ* II NEAR/4 diabet* OR typ?2 NEAR/4 diabet* OR typ?II NEAR/4 diabet* OR typ* ii NEAR/4 diabet* OR typ?ii NEAR/4 diabet* OR non insulin NEAR/4 diabet* OR noninsulin NEAR/4 diabet* OR late or adult* NEAR/4 diabet* OR matur* NEAR/4 diabet* OR slow NEAR/4 diabet* OR stabl* NEAR/4 diabet* OR ketoresist* NEAR/4 diabet* OR keto* resist* NEAR/4 diabet* OR keto* prone NEAR/4 diabet*)
#5	TI=(NIDDM OR MODY OR t2d OR typ* two NEAR/4 diabet* OR typ?two NEAR/4 diabet* OR typ* 2 NEAR/4 diabet* OR typ* II NEAR/4 diabet* OR typ?2 NEAR/4 diabet* OR typ?II NEAR/4 diabet* OR typ* ii NEAR/4 diabet* OR typ?ii NEAR/4 diabet* OR non insulin NEAR/4 diabet* OR noninsulin NEAR/4 diabet* OR late or adult* NEAR/4 diabet* OR matur* NEAR/4 diabet* OR slow NEAR/4 diabet* OR stabl* NEAR/4 diabet* OR ketoresist* NEAR/4 diabet* OR keto* resist* NEAR/4 diabet* OR keto* prone NEAR/4 diabet*)
#6	#4 OR #5
#7	#3 AND #6
#8	TS=(obese OR obesit* OR weight* NEAR/2(gain* or control* or manage* or maintain* or<br>maintenance or increas* or cycl* or fluctuation* or variation*) or overweight* or over weight* or adipos* or "body fat distribut*")
#9	TI=(obese OR obesit* OR weight* NEAR/2(gain* or control* or manage* or maintain* or<br>maintenance or increas* or cycl* or fluctuation* or variation*) or overweight* or over weight* or adipos* or "body fat distribut*")
#10	#8 OR #9
#11	#7 AND #10

© 2022 Cioana M et al. *JAMA Network Open.*

eTable 6. Characteristics of Included Studies

Source	Country	Study design	Age, years		Duration of diabetes, years	Prevalence, No. (%)	Sample size	No. (%)			Risk of bias	Level of evidence
			At diagnosis of T2DM	At study enrollment /measurement				Distribution: Sex	Distribution: Racial	Obesity prevalence by sex or racial group		
Kitagawa et al,[38] 1994	Japan	Cross-sectional	<15	<15	0	111 (85.4)	130	Male: 58 (44.6)<br>Female: 72 (55.4)	Japanese: 130 (100.0)[a]	Male: 56 (96.6)<br>Female: 55 (76.3)<br>Japanese: 111 (85.4)	Moderate	1
Pinhas-Hamiel et al,[39] 1996	United States	Cross-sectional	13.8 (1.9)	13.8 (1.9)	0	50 (92.6)	54	Male: 20 (37.0)<br>Female: 34 (63.0)	Non-Hispanic Black: 37 (68.5)<br>Non-Hispanic White: 17 (31.5)	None reported	Low	1
Scott et al,[40] 1997	United States	Cross-sectional	13.9 (0.4)[b]	13.9 (0.4)[b]	0	42 (85.7)	49	Male: 19 (38.8)<br>Female: 30 (61.2)	African American: 36 (73.5)<br>Non-Hispanic White: 12 (24.5)<br>Hispanic: 1 (2.0)	None reported	Moderate	3
Glaser et al,[41] 1998	United States	Cross-sectional	12.8 (5.0-17.0)	12.8 (5.0-17.0)	0	9 (50.0)	18	Male: 6 (33.3)<br>Female: 12 (66.7)	Mexican American: 12 (66.7)<br>Non-Hispanic White: 3 (16.7)<br>African American: 2 (11.1)<br>Cambodian: 1 (5.6)	Male: 5 (83.3)<br>Female: 4 (33.3)<br>Mexican American: 6 (50.0)<br>Non-Hispanic White: 1 (33.3)<br>African American: 1 (50.0)<br>Cambodian: 0 (0.0)	Moderate	2
Ramacha	India	Cross-	13.0 (1.8)	13.0 (1.8)	0	9 (50.0)	18	Male: 5	Indian: 18 (100.0)[a]	Male: 3	Moderate	2

© 2022 Cioana M et al. *JAMA Network Open.*

ndran et al,[42] 2003		sectional	[9-15]	[9-15]				(27.7)<br>Female: 13 (72.3)		(60.0)<br>Female: 6 (46.2)<br>Indian: 9 (50.0)		
Upchurch et al,[43] 2003	United States	Cross-sectional	13.6 (2.33)	13.6 (2.33)	0	91 (92.9)	98	Male: 48 (49.0)<br>Female: 50 (51.0)	Unknown: 42 (42.9)<br>African American: 28 (28.6)<br>Hispanic: 22 (22.4)<br>Non-Hispanic White: 3 (3.1)<br>Asian: 3 (3.1)	None reported	Moderate	3
Wei et al,[44] 2003	Taiwan	Cross-sectional	Male: 13.7 (2.5)<br>Female: 13.0 (2.5)[b]	Male: 13.7 (2.5); Female: 13.0 (2.5)[b]	0	63 (48.1)	131	Male: 50 (38.2)<br>Female: 81 (61.8)	Tamerican Indianwanese: 131 (100.0)[a]	Male: 27 (54.0)<br>Female: 36 (44.4)<br>Tamerican Indianwanese : 63 (48.1)	Low	1
Ehtisham et al,[45] 2004	United Kingdom	Cross-sectional	None reported	12.8 (3.7-15.9)[c]	0	18 (72.0)	25	Male: 7 (28.0)<br>Female: 17 (68.0)<br>Unknown: 1 (4.0)	Non-Hispanic White: 11 (44.0)<br>South Asian: 8 (32.0)<br>Other: 6 (24.0)	None reported	Low	2
Campbell-Stokes et al,[46] 2005	New Zealand	Cross-sectional	13.7 (12.1–14.8)	None reported	0	11 (92.0)	12	Male: 9 (75.0)<br>Female: 3 (25.0)	Maori: 6 (50.0)<br>European: 4 (33.3)<br>Maori/Pacific Islander: 1 (8.3)<br>European/ Fijian Indian: 1 (8.3)	None reported	Low	2
Reinehr et al,[47] 2005	Germany	Cross-sectional	14.2 (13.0-15.0)[d]	14.2 (13.0-15.0)[d]	0	14 (87.5)	16	Male: 10 (62.5)<br>Female: 6 (37.5)	Non-Hispanic White: 16 (100.0)	Non-Hispanic White: 14 (87.5)	Moderate	2
Eppens et al,[48] 2006 (Western Pacific)	Western Pacific	Cross-sectional	12.0 (10.7–13.5)[d]	14.9 (13.2–16.4)[d]	2.3 (1.4-3.6)[d]	106 (32.0)	331	Male: 149 (45.0)	Japanese: 113 (34.1)<br>South Korean: 46 (13.9)<br>Tamerican	Japanese: 43 (38.1)<br>South Korean: 8 (17.4)<br>Tamerican	Low	1

© 2022 Cioana M et al. *JAMA Network Open.*

									Indianwanese: 33 (10.0)	Indianwanese: 9 (27.3)		
									Chinese: 26 (7.9)	Chinese: 2 (7.7)		
									Malaysian: 23 (6.9)	Malaysian: 7 (30.4)		
									Filipino: 20 (6.0)	Filipino: 3 (15.0)		
									Singaporean: 20 (6.0)	Singaporean: 3 (15.0)		
									Thamerican Indian: 20 (6.0)	Thamerican Indian: 9 (45.0),		
									Hong Kongese: 13 (3.9)	Hong Kongese: 8 (61.5)		
									Australian: 10 (3.0)	Australian: 6 (60.0)		
									Aboriginal: 7 [2.1]	Indonesian: 4 (57.1)		
									White: 3 [0.9]			
									Samoan: 1 [0.3])			
								Female: 182 (55.0)	Indonesian: 7 (2.1)			
Farah et al,[49] 2006	United States	Cross-sectional	<21	10-35	1.8 (<2-15)	29 (72.5)	40	Male: 19 (47.5)	African American: 20 (50.0)	None reported	Moderate	3
									Hispanic: 13 (32.5)			
									Unknown: 3 (7.5)			
									Non-Hispanic White: 2 (5.0)			
									Filipino: 1 (2.5)			
								Female: 21 (52.5)	Indian: 1 (2.5)			
Huang et al,[50] 2006	Taiwan	Cross-sectional	11.7 (2.3)	11.7 (2.3)	0	15 (68.2)	22	Male: 6 (27.3)	Tamerican Indianwanese: 22 (100.0)[a]	Tamerican Indianwanese: 15 (68.2)	Low	2
								Female: 16 (72.7)				
Fortmeier-Saucier et al,[59] 2008	United States	Cross-sectional	None reported	8-20	None reported	44 (89.8)	49	Nr	Mexican American: 49 (100.0)	Mexican American: 44 (89.8)	Moderate	3
Lawrence et al,[51]	United States	Cross-sectional	<20	Male: 16.5 (2.8)	Male: 1.8 ± 1.7,	401 (77.1)	520	Male: 193 (37.1)	African American: 173 (33.3)	Male: 147 (76.2)	Low	1

© 2022 Cioana M et al. *JAMA Network Open.*

2008				Female: 16.1 (2.8	Female: 2.2 (1.9)			Female: 327 (62.9)	Hispanic: 114 (21.9)<br>Non-Hispanic White: 101 (19.4)<br>American Indian: 77 (14.8)<br>Asian-Pacific Islander: 40 (7.7)<br>Other/unknown: 15 (2.9)	Female: 252 (77.7)		
Bell et al,[52] 2009	United States	Cross-sectional	<20	10-14: 41 (39.0)<br>≥15: 64 (61.0)	None reported	83 (79.0)	105	None reported	Non-Hispanic White: 105 (100.0)	Non-Hispanic White: 83 (79.0)	Low	1
Liu et al,[53] 2009[e]	United States	Cross-sectional	<20	3-11: 29 (6.8)<br>12-19: 400 (93.2)	≤1: 152 (35.4), >1 - 4: 221 (51.5), >4: 58 (13.5)	331 (77.2)	429	Male: 151 (35.2)<br>Female: 278 (64.8)	African American: 133 (31.0)<br>Hispanic: 91 (21.2)<br>Non-Hispanic White: 82 (19.1)<br>American Indian: 78 (18.2)<br>Asian-Pacific Islander: 45 (10.5)	African American: 111 (83.5)<br>Hispanic: 63 (69.2)<br>Non-Hispanic White: 64 (78.0)<br>American Indian: 59 (75.6)<br>Asian-Pacific Islander: 34 (75.5)	Low	1
Liu et al,[54] 2009 (API)	United States	Cross-sectional	<20	None reported	Asian: 1.63 (1.39), Pacific Islander: 1.73 (1.73), API: 3.4 (3.06)[f]	38 (76.0)	50	None reported	Asian: 33 (66.2)<br>Asian-Pacific Islander: 10 (20.6)<br>Pacific Islander: 7 (13.2)	Asian: 23 (69.7)<br>Asian-Pacific Islander: 7 (70.0)<br>Pacific Islander: 7 (100.0)	Moderate	1
Shiga et al,[55] 2009	Japan	Cross-sectional	None reported	16.5 (3.5)	3.8 (2.8)	28 (65.1)	43	Male: 15 (35.0)<br>Female: 28 (65.0)	Japanese: 43 (100.0)[a]	Male: 13 (86.7)<br>Female: 15 (53.6);<br>Japanese: 28 (65.1)	Moderate	3
Urakami	Japan	Cross-	12.9 (1.5)	12.9 (1.5)	0	93 (83.0)	112	Male: 45	Japanese: 112 (100.0)[a]	Japanese: 93	Moderate	3

© 2022 Cioana M et al. *JAMA Network Open.*

et al,[56] 2009		sectional						(40.2)<br>Female: 67 (59.8)		(83.0)		
Amed et al,[57] 2012	Canada	Cross-sectional	Aboriginal: 12.9 (12.4-13.4)[g]<br>White: 14.4 (13.8-15.1)[g]<br>Other ethnicity: 14.3 (13.7-14.9)[g]	Aboriginal: 12.9 (12.4-13.4)[g]<br>White: 14.4 (13.8-15.1)[g]<br>Other ethnicity: 14.3 (13.7-14.9)[g]	0	211 (95.5)	221	Male: 91 (41.2)<br>Female: 130 (58.8)	Canadian Aboriginal: 100 (45.2)<br>Non-Hispanic White: 57 (25.8)<br>Other (African/Caribbean, Asian, Hispanic, Middle Eastern): 64 (29.0)	Canadian Aboriginal: 92 (92.0)<br>Non-Hispanic White: 56 (98.2)<br>Other: 63 (98.3)	Moderate	3
Zabeen et al,[58] 2016	Bangladesh	Cross-sectional	9-10: 11 (14)<br>11-14: 46 (60)<br>15-17: 20 (26)	9-10: 11 (14)<br>11-14: 46 (60)<br>15-17: 20 (26)	0	45 (58.4)	77	Male: 26 (33.8)<br>Female: 51 (66.2)	Bangladeshi: 77 (100.0)[a]	Bangladeshi: 45 (58.4)	Low	1
Alsaffar et al,[61] 2020	Iraq	Cross-sectional	14.8 (2.9)	14.8 (2.9)	0	16 (100.0)	16	Male: 10 (62.5)<br>Female: 6 (37.5)	Iraqi: 16 (100)[a]	Male: 10 (100)<br>Female: 6 (100)<br>Iraqi: 16 (100)	Low	2
Ludwig et al,[60] 2021	Australia And New Zealand	Cross-sectional	13.7 (12.0-14.9)[d,f]	14.3 (12.7-15.6)[d,f]	0.2 (0.0-0.8)[d,f]	199 (76.5)	260	Male: 113 (42.0)<br>Female: 156 (58.0)[f]	Non-Indigenous: 137 (57.6)<br>Indigenous Australian: 51 (23.1)<br>Maori/Pacific Islander: 46 (19.3)	Non-Indigenous: 100 (73.0)<br>Indigenous Australian: 33 (64.7<br>Maori/Pacific Islander: 43 (93.5)	Low	1
Shilbayeh et al,[62] 2021	Saudi Arabia	Cross-sectional	13.90 (5.0)	18.45 (6.5)	None reported	38 (77.6)	49	Male: 22 (44.9)<br>Female: 27 (55.1)	Saudi: 49 (100)	Saudi: 38 (77.6)	Low	2
Xu et al,[63] 2021	China	Cross-sectional	12.4 (1.8) (6.6-16.7)[f]	12.4 (1.8)[f]	None reported	89 (58.2)	153	Male: 93 (57.8)<br>Female: 68	Chinese: 161 (100)[f]	Chinese: 89 (58.2)	Low	1

© 2022 Cioana M et al. *JAMA Network Open.*

								(42.2)[f]				
Dean et al,[64] 1992	Canada	Retrospective cohort	12.1 (7-14)	12.1 (7-14)	0	9 (45.0)	20	Male: 4 (20.0)<br>Female: 16 (80.0)	Canadian Aboriginal: 20 (100.0)	Canadian Aboriginal: 9 (45.0)	Low	2
Coddington et al,[65] 2001	United States	Retrospective cohort	13.7 (7.0-20.0)	13.7 (7.0-20.0)	0	18 (81.8)	22	Male: 10 (45.5)<br>Female: 12 (54.5)	American Indian: 22 (100.0)	American Indian:18 (81.8)	Low	2
Grinstein et al,[66] 2003	United States	Retrospective cohort	14.0 (2.3)	14.0 (2.3)	0	>70%	83	Male: 31 (37.3)<br>Female: 52 (62.7)	African American: 43 (51.8)<br>Caribbean Hispanic: 37 (44.6)<br>American Indian: 3 (3.6)	None reported	Moderate	3
Zdravkovic et al,[67] 2004	Canada	Retrospective cohort	13.5 (2.2) [8.8-17.5]	13.5 (2.2) [8.8-17.5]	0	33 (80.5)	41	Male: 15 (36.6)<br>Female: 26 (63.4)	South/East Asian: 19 (46.3)<br>African Canadian: 11 (26.8)<br>Non-Hispanic White: 6 (14.6)<br>Hispanic: 4 (9.8)<br>Fn: 1 (2.4)	South/East Asian: 14 (73.7)<br>African Canadian: 9 (81.8)<br>Non-Hispanic White: 5 (83.3)<br>Hispanic: 3 (75.0)<br>Fn: 1 (100.0)	Low	2
Scott et al,[68] 2004	New Zealand	Retrospective cohort	None reported	19.6 (14-23)	1.7	13 (100.0)	13	Male: 7 (53.8)<br>Female: 6 (46.2)	Maori: 7 (53.8)<br>European: 4 (30.8)<br>Pacific Islander: 1 (7.7)<br>Asian Indian: 1 (7.7)	None reported	Moderate	2
Pérez-Perdomo et al,[69] 2005	Puerto Rico	Retrospective cohort	14 (2.7)[f]	None reported	None reported	69 (80.2)	86	Male: 27 (31.2)<br>Female: 59 (68.8)	None reported	None reported	Moderate	1
Sugihara et al,[70] 2005	Japan	Retrospective cohort	11.9 (2.1) [6-16]	11.9 (2.1) [6-16]	0	179 (69.9)	256	Male: 119 (46.5)<br>Female:	Japanese: 256 (100.0)[a]	Male: 93 (78.2)<br>Female: 86 (62.8)<br>Japanese: 179	Low	1

© 2022 Cioana M et al. *JAMA Network Open.*

								137 (53.5)		(69.9)		
Sellers et al,[71] 2007	Canada	Retrospective cohort	13.1 (9–17)	15.3 (9-18)	None reported	38 (38.4)	99	Male: 42 (42.5)<br>Female: 57 (57.5)	Fn/Metis: 94 (94.9)<br>Other: 5 (5.1)	Male: 18 (42.9)<br>Female: 19 (33.3)	Low	1
Balasanthiran et al,[72] 2012	United Kingdom	Retrospective cohort	15.2 (3.34)	21.2 (3.19)	5.4 (3.09)	23 (59.0)[h]	39	Male: 15 (38.5)<br>Female: 24 (61.5)	Bangladeshi: 10 (25.6)<br>Pakistani: 8 (20.5)<br>Indian: 6 (15.9)<br>White British: 5 (13.6)<br>Black African: 4 (10.3)<br>Black Caribbean: 4 (10.3)<br>Unknown: 2 (5.1)[h]	None reported	Moderate	2
Fu et al,[73] 2013	China	Retrospective cohort	<18	<10: 62 (17.8)<br>10-18: 287 (82.2)	None reported	248 (71.1)	349	Male: 202 (57.9)<br>Female: 147 (42.1)	Chinese: 349 (100.0)[a]	Chinese: 248 (71.1)	Low	1
Osman et al,[74] 2013	Sudan	Retrospective cohort	<11: 3 (7.9),<br>11-18: 35 (92.1)	None reported	None reported	29 (76.3)	38	Male: 17 (44.7)<br>Female: 21 (55.3)	Arab: 32 (84.2)<br>Mixed: 4 (10.5)<br>Non-Arab: 2 (5.3)	None reported	Low	2
Haynes et al,[75] 2014	Australia	Retrospective cohort	13.3 (2.0)	13.3 (2.0)	0	82 (60.7)	135	Male: 53 (39.3)<br>Female: 82 (60.7)	Indigenous: 76 (56.3)	None reported	Moderate	1
Newton et al,[76] 2015	New Zealand	Retrospective cohort	6.5-17	<17	None reported	22 (95.7)	23	None reported	Samoan: 6 (26.1)<br>Maori: 4 (17.4)<br>Tokelauan: 4 (17.4)<br>Cook Island Maori: 2 (8.7)<br>Tongan: 2 (8.7)<br>Chinese: 2 (8.7)<br>Fijian: 1 (4.3)<br>Indian: 1 (4.3)<br>New Zealand European: 1 (4.3)	None reported	Moderate	2
Abbasi et al,[77] 2017	United Kingdom	Retrospective cohort	<25	<25	0	308 (47.1)	654	Male: 262 (40.1)<br>Female:	None reported	None reported	Low	1

© 2022 Cioana M et al. *JAMA Network Open.*

								392 (59.9)				
Morrison et al,[78] 2018	United Kingdom	Retrospective cohort	13.5 (8.2-17.1)	Nr	None reported	9 (50.0)	18	Male: 5 (27.8)<br>Female: 13 (72.2)	South Asian: 15 (83.3)<br>Other: 3 (16.7)	None reported	Moderate	3
Greenup et al,[80] 2020	United States	Retrospective cohort	5-8: 8 (19.0)<br>9-10: 34 (81.0)	5-8: 8 (19.0)<br>9-10: 34 (81.0)	0	40 (95.2)	42	Male: 5 (11.9)<br>Female: 37 (88.1)	African American: 37 (88.1)<br>White: 5 (11.9)	None reported	Moderate	3
Van Name et al,[79] 2020	United States	Retrospective cohort	13.7 (12.2–15.4)[d]	13.7 (12.2–15.4)[d]	0	909 (91.1)	998	None reported	None reported	None reported	Moderate	3
Astudillo et al,[81] 2021	United States	Retrospective cohort	13.6 (2.5[f]	None reported	None reported	295 (88.6)	333	Male: 128 (34.0)<br>Female: 248 (66.0)[f]	Hispanic: 225 (59.8)<br>Black: 103 (27.4)<br>Non-Hispanic White: 26 (6.9)<br>Other: 13 (3.5)<br>Unknown: 9 (2.4)[f]	None reported	Low	1
Marks et al,[82] 2021	United States	Retrospective cohort	≤21	≤21	0	126 (73.7)	171	Male: 125 (51.0)<br>Female: 120 (49.0)[f]	Non-Hispanic Black: 167 (68.2)<br>Other: 57 (23.3)<br>Latinx: 52 (21.2)[J]<br>Non-Hispanic White: 12 (4.9)<br>Asian: 4 (1.6)<br>Unknown: 5 (2.0)[f]	None reported	Moderate	1
Tung et al,[83] 2021	Hong Kong	Retrospective cohort	14.7 (2.1)	14.7 (2.1)	0	308 (78.7)	391	Male: 203 (51.9)<br>Female: 188 (48.1)	Chinese: 161 (100)	None reported	Low	1
Kim et al,[84] 2022	United States	Retrospective cohort	None reported	16.2 (2.1)[f]	2.5 (1.5,4.7)[d,f]	237 (80.1)	296	Male: 102 (34.2)	Hispanic White: 170 (57.8)<br>Non-Hispanic Black: 86 (29.3)<br>Non-Hispanic White: 20 (6.8)<br>Hispanic Other: 8 (2.7)<br>Non-Hispanic Other: 7 (2.4)<br>Hispanic Black: 3 (1.0)	None reported	Low	1

© 2022 Cioana M et al. *JAMA Network Open.*

								Female: 196 (65.8)[E]	Unknown: 4 (1.3)[f]			
Schmitt et al,[85] 2022	United States	Retrospective cohort	13.8 (2.5)[f]	13.8 (2.5)[f]	0	474 (77.1)	615	Male: 258 (40.2)<br>Female: 384 (59.8)[f]	Non-Hispanic Black 459 (71.5)<br>Non-Hispanic White: 134 (20.1)<br>Hispanic: 42 (6.5)<br>Other 7[f]	None reported	Low	1
Zuckerman Levin,[86] 2022	Israel	Retrospective cohort	14.7 (1.9)[f]	14.7 (1.9)[f]	None reported	278 (77.2)	360	Male: 151 (39.8)<br>Female: 228 (60.2)[f]	Israeli Jews: 221 (58.3)<br>Israeli Arabs: 158 (41.7)[f]	None reported	Low	1
Eppens et al,[11] 2006 (Australia)	Australia	Prospective cohort	13.2 (11.6-15.0)[d]	15.3 (13.6-16.4)[d]	1.3 (0.6-3.1)[d]	36 (56.3)	64	Male: 32 (50.0)<br>Female: 32 (50.0)	Australian (White): 20 (31.3)<br>South Central and North Asian: 17 (26.6)<br>Aboriginal/Torres Stramerican Indiant Islander: 10 (15.6)<br>North African/Middle Eastern: 6 (9.4)<br>Southern And Eastern European: 6 (9.4)<br>Polynesian: 4 (6.3)	None reported	Moderate	1
Reinehr et al,[87] 2008	Germany and Austria	Prospective cohort	13.4 (11.8-15.1)[d]	13.4 (11.8-15.1)[d]	0	85 (65.9)	129	Male: 32 (24.8)<br>Female: 97 (75.2)	German/Austrian: 102 (79.1)	None reported	Moderate	1
Shield et al,[88] 2009	United Kingdom and Republic of Ireland	Prospective cohort	13.6 (9.9-16.8)	13.6 (9.9-16.8)	0	61 (80.3)	76	Male: 34 (44.7)<br>Female: 42 (55.3)	Non-Hispanic White: 43 (56.6)<br>South Asian: 14 (18.4)<br>African American: 13 (17.1)<br>Mixed/Chinese: 6 (7.9)	None reported	Low	1
Ruhayel et al,[89] 2010	Australia	Prospective cohort	13.4 (9.2–17.4)[c]	13.4 (9.2–17.4)[D]	0	23 (69.7)	33	Male: 11 (33.3)	Oceanian/ European: 20 (60.6) (Aboriginal/ Torres Stramerican Indiant/South Seas: 3 [9.1])	Male: 9 (81.8)	Moderate	2

© 2022 Cioana M et al. *JAMA Network Open.*

								Female: 22 (66.7)	Chinese Asians: 6 (18.2)<br>Arabic/Middle Eastern: 6 (18.2)<br>South-East Asian: 1 (3.0)	Female: 14 (63.6)		
Larkin et al,[90] 2015	United States	Prospective cohort	10-17	14.0 (2.0)	<2	605 (88.3)	685	Male: 240 (35.0)<br>Female: 445 (65.0)	Hispanic: 271 (39.6)<br>Non-Hispanic Black: 223 (32.5)<br>Non-Hispanic White: 141 (20.6)<br>Other: 50 (7.3)	None reported	Moderate	3
Guven et al,[91] 2016	Turkey	Prospective cohort	<18	Male: 11.8 (2.2) [9.1-16.9]<br>Female: 13.6 (1.7) [8.9-17.3]	None reported	53 (63.1)	84	Male: 26 (31.0)<br>Female: 58 (69.0)	Turkish: 84 (100.0)	Male: 21 (80.7)<br>Female: 32 (55.1);<br>Turkish: 53 (63.1)	Moderate	3
Candler et al,[3] 2018	United Kingdom and Republic of Ireland	Prospective cohort	14.3 (7.9-16.9)[c]	14.3 (7.9-16.9)[c]	0	86 (81.1)	106	Male: 35 (33.0)<br>Female: 71 (67.0)	Non-Hispanic White: 47 (44.3)<br>Asian/Asian-British: 36 (34.0)<br>Black: 14 (13.2)<br>Uncertamerican Indiann: 4 (3.8)<br>Other: 5 (4.7)	None reported	Low	1
Carino et al,[92] 2021	Canada	Prospective cohort	None reported	14.9 (2.3)	2.3 ± 2.0)	232 (72.2)	322	Male: 110 (34.2)<br>Female: 212 (65.8)	Indigenous: 279 (86.8)<br>Non-Hispanic White: 15 (4.7)<br>Asian-Pacific Islander: 16 (5.0)<br>Afro-Caribbean: 10 (3.1)<br>Hispanic: 1 (0.3)	None reported	Low	1

Abbreviations: FN, first nations; T2DM, type 2 diabetes mellitus.
[a]Racial distribution assumed to match country of origin.
[b]Mean (SE).
[c]Median (range).

© 2022 Cioana M et al. *JAMA Network Open.*

[d]Median (IQR).
[e]Only prevalence by racial group data from Liu.
[f]Value from total cohort instead of only from patients tested for obesity.
[g]Mean (CI).
[h]Estimated based on graph.
2009 was used for the meta-analysis because this study included the same cohort of patients as Lawrence 2008. Ages and duration reported as range.

© 2022 Cioana M et al. *JAMA Network Open.*

eTable 7. Symptoms at Presentation and Risk Factors of Patients in Included and Type 2 Diabetes Diagnostic Criteria Used Across Studies

Author, Year (Country)	Sample Size (n)	Clinical Presentation	Puberty	Diagnostic Criteria	Exclusion Criteria	Antibody Testing	Risk Factors for T2DM	Therapy at Baseline
Abbasi, 2017 (UK) [2]	654	NR	NR	Prescription of oral glucose-lowering medications only, or HbA1c ≥6.5% but no insulin prescription	Unclassifiable diabetes, prescriptions for both oral glucose-lowering medications and insulin, T1DM without an insulin prescription, and T2DM with a prescription for insulin	NR	NR	NR
Alsaffar, 2020 (Iraq) [3]	16	Acanthosis nigricans (n=16), High blood glucose (n=14), Weight gain (n=4), High C peptide (n=13), Polyuria (n=1)	NR	ADA criteria[4]	NR	IAA, ICA, GAD65: all negative	Family history of T2DM (n=16)	NR
Amed, 2012 (Canada) [5]	221	Acanthosis nigricans (n=161), DKA (n=22)	NR	CDA criteria:[6] at least one of the following: 1) random plasma glucose ≥11.1 mmol/l and presence of classical symptoms; or 2) fasting plasma glucose ≥7.0 mmol/l random plasma; or 3) 2-hour plasma glucose ≥11.1 mmol/l in response to OGTT Absence of pancreatic antibodies (when available); diagnosis supported by clinical features including obesity, a positive family history of T2DM, a history of exposure to diabetes in utero, evidence of insulin resistance, and belonging to a high-risk ethnic group	Positive pancreatic antibodies	Unspecified: all negative (only done in unclear cases)	NR	NR
Astudillo, 2021 (USA) [7]	333	Acanthosis nigricans (n=339), Diabetic ketoacidosis (n=24)[b]	Prepubertal (n=35), Pubertal (n=341)	ADA criteria[4]	NR	GAD65, IA2/ICA512, IAA ZnT8: all negative	Family history of T2DM (n=336)[b]	Insulin (n=163), metformin (n=123), insulin + metformin (n=52), lifestyle (n=37), other (n=1)[b]

© 2022 Cioana M et al. *JAMA Network Open.*

Balasanthiran, 2012 (UK) [8]	39	Acanthosis nigricans frequently noted, Weight loss and osmotic symptoms (n=7), Asymptomatic (n=5), Ketonuria (n=3), Chest pain (n=1), Vaginal candidiasis (n=1), Diabetic ketoacidosis (n=1), Unclear symptoms (n=28)	NR	NR	MODY features (not markedly obese, diabetic family members of normal weight, no acanthosis nigricans, ethnic background from a low prevalence T2DM race, no evidence of insulin resistance with fasting C-peptide in the normal range), positive ICA and GAD antibodies	Unspecified: all negative (only done in unclear cases)	Family history of T2DM: at least one parent (n=22), sibling (n=4), 2nd-degree relative (n=28)	NR
Bell, 2009 (USA) [9]	105	Acanthosis nigricans (n=32), DKA (n=4)	NR	ADA criteria,[10] T2DM diagnosed in patients with negative pancreatic antibodies and fasting C-peptide ≥3.7 ng/ml or using clinical definitions	MODY, hybrid, other types, or missing diabetes type	GAD: positive (n=21)	Family history of T2DM (n=88)	Metformin only (n=53), insulin only (n=23), insulin + metformin (n=26), no medication (n=6)[b]
Campbell-Stokes, 2005 (New Zealand) [11]	12	Acanthosis nigricans (n=8)	Pubertal (n=9)	ADA criteria[4]: at least one of the following:<br>1) random plasma glucose ≥11.1 mmol/l and presence of classical symptoms; or<br>2) fasting plasma glucose ≥7.1 mmol/l; or<br>3) 2-hour plasma glucose ≥11.1 mmol/l in response to OGTT	Diabetes secondary to stress or drugs	Unspecified: negative (n=8), positive (n=2), data not available (n=2)	Family history of T2DM: 1st-degree relative (n=5), 2nd-degree relative (n=1)	Oral hypoglycemic agent (n=4), insulin (n=5), insulin + oral hypoglycemic agent (n=3)
Candler, 2018 (UK and Republic of Ireland) [12]	106	Ketonuria (n=53), Asymptomatic (n=37), Osmotic symptoms (polyuria, polydipsia, nocturia, weight loss) (n=37), Recurrent infection (n=19), Lethargy (n=16), DKA/HHS (n >5)	NR	ADA criteria[4] or HbA1c >6.5%. T2DM was distinguished from other types of diabetes based on:<br>1) presence of raised insulin level (>132 pmol/l) or raised C-peptide level (>0.6 nmol/l); or<br>2) the child was managed off insulin therapy for >9 months in the absence of typical T1DM autoantibodies	T1DM (positive antibodies and/or persisting insulin requirement from diagnosis), MODY, diabetes-associated syndrome (Prader–Willi or Bardet–Biedl syndromes), diabetes secondary to medication, pancreatic failure	Unspecified: all negative (only done in unclear cases)	Family history of T2DM (n=86): 1st-degree relative (n=74), 2nd-degree relative (n=12)	NR

Carino, 2021 (Canada) [13]	322	NR	NR	CDA criteria[6]	Cancer history, high-dose steroids or immunosuppressive medications, concomitant chronic inflammatory conditions, alcohol or drug use, pregnancy, and inability or unwillingness to provide consent	Unspecified: all negative	NR	NR
Coddington, 2001 (USA) [14]	22	Polyuria, nocturia, enuresis, weight loss, fatigue, or recurrent skin infections (n=11), DKA (n=1)	NR	National Diabetes Data Group criteria[15]: patient<br>1) met OGTT or random glucose (two values >11 mmol/L) criteria for diabetes;<br>2) was not ketosis prone under basal conditions;<br>3) did not require exogenous insulin to prevent diabetic ketoacidosis for extended periods (1 to 11 years in this population); and<br>4) did not have illnesses or medications predisposing them to the development of secondary diabetes	NR	NR	Family history of T2DM: 1st-degree relative (n=19); maternal gestational diabetes (n=11); large for gestational age (n=2)	Oral hypoglycemic agent (n=14), insulin (n=5), diet alone (n=3)
Dean, 1992 (Canada) [16]	20	Asymptomatic (n=15), polyuria/nocturia (n=5), ketonuria (n=5)	NR	NR	NR	ICA: negative (n=10/14), weakly positive (n=2/14), positive (n=2/14)	Family history of T2DM: 1st-degree relative (n=16)	Insulin + diet + exercise counselling (n=4), diet + exercise program (n=16)
Ehtisham, 2004 (UK) [17]	25	Ketonuria (n=6)	Pubertal (n=19), prepubertal (n=6)	WHO criteria[18]: idiopathic non-syndromic insulin resistance (with evidence of acanthosis nigricans, raised insulin or C-peptide, or abnormal lipid profile)	MODY, diabetes-associated syndromes, patients treated with insulin, T1DM	GAD positive (n=1); Unspecified: transiently raised (n=1)	Family history of T2DM: 1st-degree relative (n=14), other relatives (n=7)	Oral hypoglycemic agent (n=18), insulin (n=2), insulin + oral hypoglycemic agent (n=5)
Eppens, 2006 (Australia) [19]	64	NR	NR	Australasian Pediatric Endocrine Group criteria[20]: negative diabetes-associated antibodies, elevated fasting insulin or C-peptide, or presence of acanthosis nigricans	Positive T1DM-associated autoantibodies	Unspecified: all negative (n=34/34)	NR	Oral hypoglycemic agents or diet/ exercise or insulin (n=NR)

© 2022 Cioana M et al. *JAMA Network Open.*

Eppens, 2006 (Western Pacific) [21]	331	Acanthosis nigricans (n=111)	NR	OGTT, C-peptide or insulin levels, negative diabetes-associated autoantibodies, or clinical judgement	Positive T1DM-associated autoantibodies	Unspecified: all negative (only done in unclear cases)	Family history of T2DM: at least one parent (n=146), sibling (n=26)	Oral hypoglycemic agent (n=162) (biguanide [n=87], sulphonylurea [n=23]), insulin only (n=36), oral hypoglycemic agent + insulin (n=51), no medication (n=82)
Farah, 2006 (USA) [22]	40	Polyuria (n=18), Asymptomatic (n=15), Polydipsia (n = 12), Weight loss (n = 3)	Pubertal (n=40)	NR	NR	NR	Family history of T2DM (n=35)	Oral hypoglycemic agent (n=24), insulin (n=6), oral hypoglycemic agent + insulin (n=6), diet alone (n=3)
Fortmeier-Saucier, 2008 (USA) [23]	49	NR	NR	Diagnosis confirmed by laboratory testing	NR	NR	NR	NR
Fu, 2013 (China) [24]	349	NR	NR	WHO[18] and ADA criteria[25], using OGTT	NR	NR	NR	NR
Glaser, 1998 (USA) [26]	18	Acanthosis nigricans (n=12), Ketonuria (n=5), Acidosis (n=2)	NR	1) abnormal insulin secretion following the diagnosis of diabetes mellitus, as determined either by the measurement of C-peptide or insulin concentrations or by successful treatment with dietary management with or without oral hypoglycemic agents for more than two years after diagnosis; and<br>2) the absence of ICA or insulin autoantibodies at the time of the diagnosis.	positive ICA	ICA: all negative	Family history of T2DM (n=13)	Oral hypoglycemic agent (n=11), insulin (n=3), oral hypoglycemic agent + insulin (n=1), diet alone (n=3)

© 2022 Cioana M et al. *JAMA Network Open.*

Greenup, 2020 (USA) [27]	42	Polyuria, polydipsia, or polyphagia (n=32), Vaginal candidiasis (n=7), Weight loss (n=5)	Pubertal (n=34), prepubertal (n=8) (assumed)	Clinical diagnosis of T2DM from medical records and HbA1c ≥6.5%	T1DM, MODY, cystic fibrosis-related diabetes, chronic renal or pancreatic disease, Prader-Willi syndrome, or conditions requiring chronic systemic steroid use or immunosuppression	GAD65: positive (n=4/39)[a]	Family history of T2DM (n=42): 1st-degree relative (n=30); low birth weight (< 2500g) (n=6); high birth weight (>4000g) (n=5); born prematurely (n=4)	Metformin (n=10), insulin (n=8), insulin + metformin (n=24)
Grinstein, 2003 (USA) [28]	83	Acanthosis nigricans (n=74), Polyuria and polydipsia (n=38), Ketonuria (n=31), Asymptomatic (n=25), Weight loss (n=18), Fatigue (n=11), DKA (n=5)	Pubertal (n=89)	National Diabetes Data Group criteria[15]	positive ICA, insulin and/or GAD antibodies	ICA, insulin and/or GAD: all negative	Family history of T2DM: 1st-degree relative (n=58), 2nd-degree relative (n=45)	Oral hypoglycemic agent (metformin and/or glipizide) (n=74), insulin (n=12), insulin + oral hypoglycemic agent (n=8), no medication (n=6)
Guven, 2016 (Turkey) [29]	84	NR	NR	NR	NR	Unspecified: positive (n=15/60)	NR	NR
Haynes, 2014 (Australia) [30]	135	NR	NR	NR	NR	NR	NR	NR
Huang, 2006 (Taiwan) [31]	22	Acanthosis nigricans (n=14)	NR	ADA criteria[4]	T1DM, secondary causes of diabetes or diabetes-associated syndromes (hemochromatosis, congenital generalized lipodystrophy, Wolfram syndrome, Prader-Willi syndrome, or Turner syndrome)	GAD and IA-2: all negative	NR	NR

© 2022 Cioana M et al. *JAMA Network Open.*

Kim, 20221 (USA) [32]	296	NR	NR	NR	Statin therapy at the time of most recent LDL-C concentration, inability to calculate or directly measure LDL-C at the lab or lack of LDL-C screening	NR	NR	Insulin (n=159), other (n=139)[b]
Kitagawa, 1994 (Japan) [33]	130	NR	NR	Urine screening, followed by OGTT and confirmation of diabetes type with serum ICA and several months or more of observation of glucose intolerance and pancreatic beta cell function	Positive antibodies, failure to improve glucose tolerance in spite of strict dietary control and physical exercise, insulin therapy within 18 months of diagnosis	ICA: all negative	NR	NR
Larkin, 2015 (USA) [34]	685	NR	NR	ADA criteria[25] and negative pancreatic autoantibodies	Positive pancreatic antibodies	Unspecified: all negative	NR	Metformin (n=225), metformin + rosiglitazone (n=232), metformin + lifestyle program (n=227)
Lawrence, 2008 (USA) [35]	520	NR	NR	ADA criteria,[10] T2DM diagnosed in patients with negative pancreatic antibodies and fasting C-peptide ≥3.7 ng/ml or using clinical definitions	MODY, hybrid, other types, or missing diabetes type	GAD, IA-2, insulin: results NR	NR	NR
Liu, 2009 (USA) [36]	429	NR	NR	ADA criteria,[10] T2DM diagnosed in patients with negative pancreatic antibodies and fasting C-peptide ≥3.7 ng/ml or using clinical definitions	MODY, hybrid, other types, or missing diabetes type	GAD, IA-2, insulin: results NR	NR	Metformin (n=180), insulin (n=82), insulin + metformin (n=64), no medication (n=47), missing (n=56)
Liu, 2009 (API) (USA) [37]	50	DKA (n=2)	NR	ADA criteria,[10] T2DM diagnosed in patients with negative pancreatic antibodies and fasting C-peptide ≥3.7 ng/ml or using clinical definitions	MODY, hybrid, other types, or missing diabetes type	GAD65: positive (n = 7)	Family history of T2DM (n=36)	Metformin (n=31), insulin (n=7), metformin + insulin (n=4), no medication (n=7)
Ludwig, 2021 (Australia and New Zealand) [38]	260	NR	NR	ISPAD guidelines[39]: fasting glucose, 2-hour plasma glucose in response to OGTT or HbA1c; in the absence of symptoms, testing should be confirmed with a repeat test on a different day	MODY assessed through genetic testing	Unspecified: all negative	NR	NR
Marks, 2021 (USA)[40]	171	DKA (n=39), Weight loss (n=15), Hyperosmolar DKA (n=13)[b]	NR	ADA criteria[4]; T2DM was diagnosed in obese (BMI Z-score >1.64) patients in the absence of pancreatic autoantibodies and DKA	Strong clinical suspicion or positive genetic testing for monogenic diabetes (n=7)	GAD65, islet antigen 2, insulin, ZnT8, ICA: all negative	NR	NR

© 2022 Cioana M et al. *JAMA Network Open.*

Morrison, 2018 (UK) [41]	18	NR	NR	NR	NR	NR	NR	Metformin (n = 17), insulin (n=4)
Newton, 2015 (New Zealand) [42]	23	NR	NR	NR	NR	NR	NR	NR
Osman, 2013 (Sudan) [43]	38	Acanthosis nigricans (n=31), Polyuria, polydipsia and/or weight loss (n=27), Asymptomatic (n=11), Stunted growth (n=4)	Pubertal (n=35)	Clinical diagnosis based on T2DM symptoms at onset, presence of obesity, acanthosis nigricans, other features of metabolic syndrome, family history of T2DM and availability of abnormal insulin, or C-peptide levels as well as treatment given	NR	NR	Family history of T2DM (n=35), large for gestational age (n=16), small for gestational age (n=12)	Metformin (n=18), metformin + insulin (n=8), glibenclamide (n = 1), metformin + insulin + glibenclamide (n=3), no medication (n=2)
Pérez-Perdomo, 2005 (Puerto Rico) [44]	86	Acanthosis nigricans (n=45), Polyuria (n=30), Polydipsia (n=25), Consistent hunger (n=15), Headaches (n=13)	NR	NR	NR	NR	Family history of T2DM (n=73)	Oral hypoglycemic agent (n=38), insulin (n=6), oral hypoglycemic agent + insulin (n=16)
Pinhas-Hamiel, 1996 (USA) [45]	54	Polyuria, polydipsia, and/or weight loss for a few months before presentation (almost all patients), Acanthosis nigricans (n=32), Vaginal monilial infection (n=9), Severe infection (n=2), Dysuria, enuresis, and/or abdominal pain (some patients)	Mid puberty (Tanner stage III or greater) (n=53), prepubertal (n=1)	National Diabetes Data Group criteria[15]	MODY	ICA: all negative	Family history of T2DM (n=46): 1st-degree relative (n=35)	NR
Ramachandran, 2003 (India) [46]	18	Asymptomatic (n=9), Polyuria and polydipsia (n=5), Acanthosis nigricans (n=4), Ketonuria (n=1)	NR	1) insidious onset of diabetes at age of less or equal to 15 years; 2) response to treatment with oral antidiabetic agents; 3) presence of insulin secretory capacity comparable to T2DM in adults, as indicated by serum C-peptide concentrations (stimulated response ≥0.6 pmol/ml); and	Positive GAD65 antibodies	GAD65: all negative	Family history of T2DM (n=18): 1st-degree relative (n=16), 2nd-degree family (n=2)	Oral hypoglycemic agent (n=12) (metformin [n=5], hlipizide [n=2], glyclazide [n=2], hlibenclamide [n=1], metformin + glibenclamide [n=2]), insulin + oral hypoglycemic agent (n=6) (insulin + metformin [n=1], insulin + glipizide [n=1], insulin + glibenclamide [n=1], insulin + metformin + glibenclamide [n=1], insulin + metformin +

© 2022 Cioana M et al. *JAMA Network Open.*

				4) negative GAD65 antibodies				glyclazide [n=2]), enalpril (n=1)
Reinehr, 2005 (Germany) [47]	16	Asymptomatic (n=13), Acanthosis nigricans (n=8), Polyuria and polydipsia (n=3), Ketonuria/ketoacidosis (n=0)	Pubertal (n=16)	ADA criteria[4]	Positive pancreatic autoantibodies	Unspecified: all negative	Family history of T2DM (n=12)	Oral hypoglycemic agent (n=9) (metformin [n=8], sulfonylurea [n=1]), insulin + oral hypoglycemic agent (n=5)
Reinehr, 2008 (Germany and Austria) [48]	129	Ketoacidosis (n=5), Ketonuria (n=3)	NR	ADA[4] and ISPAD criteria[49], obesity, pubertal age and acanthosis nigricans, slow and mild manifestation and no insulin dependence support diagnosis; if initial insulin treatment, T2DM was diagnosed only if no beta cell and insulin antibodies were detected and if insulin deficiency was ruled out by C-peptide values, or if initial insulin treatment could be stopped for at least 1 year	Initial insulin dependence, MODY, genetic syndromes, and secondary diabetes	Unspecified: all negative	NR	Oral hypoglycemic agent (n=18) (metformin [n=12], sulfonyl urea [n=4], glinide [n=1], insulin sensitizer [n=1]), insulin (n=40), insulin + metformin (n=6), lifestyle (n=65)
Ruhayel, 2010 (Australia) [50]	33	NR	NR	ISPAD criteria[51], clinical details used such as strong family history of T2DM, being of high-risk ethnicity and presence of acanthosis nigricans to support diagnosis	Positive ICA, GAD, and insulin antibodies	GAD, insulin and ICA: all negative	NR	Oral hypoglycemic agent (n=17) (metformin [n=15], metformin + glibenclamide [n=2]), insulin (n=5), metformin + insulin (n=9)
Schmitt, 2022 (USA)[52]	642	NR	NR	T2DM defined according to the presence of any ICD-10 code consistent with T2D (E11.x). If a patient had codes for both type 1 diabetes (E10.x) and T2D (E11.x), manual chart review was performed to determine the relevant diagnosis based upon clinical phenotype, antibody status, insulin use, and provider documentation	NR	NR	NR	NR

© 2022 Cioana M et al. *JAMA Network Open.*

Scott, 1997 (USA) [53]	49	Acanthosis nigricans (n=42), Polyuria (n=35), Polydipsia (n=34), Nocturia (n=26), Polyphagia (n=24), Headache (n=17), Weight loss (n=16), Abdominal pain (n=13), Dizziness (n=13), Visual disturbance (n=8)	NR	National Diabetes Data Group[15] and WHO criteria[18], negative antibody testing to confirm the diagnosis if needed	Positive pancreatic antibodies	Unspecified: all negative (only done in unclear cases)	NR	NR
Scott, 2004 (New Zealand) [54]	13	NR	NR	Diagnostic blood sugars with negative antibodies or no history of ketoacidosis	Positive IA-2 and GAD antibodies	IA-2 and GAD: all negative	NR	Metformin (n=4), insulin (n=1), insulin + metformin (n=2), insulin + acarbose (n=1), diet alone (n=6)
Sellers, 2007 (Canada) [55]	99	NR	NR	CDA criteria[56], supported by First Nations heritage, first degree family history of T2DM, obesity, and the presence of acanthosis nigricans. Negative ICA, GAD and insulin antibodies used in cases where the classification was uncertain	Positive ICA, GAD and insulin antibodies	ICA, GAD, insulin: all negative (only done in unclear cases)	NR	NR
Shield, 2009 (UK and Republic of Ireland) [57]	76	Ketosis (n=10)	NR	ADA criteria,[4] and raised fasting insulin (>132 pmol/l) or fasting C-peptide concentrations (>0.6 nmol/l) and/or negative ICA, GAD, insulin antibodies, with no insulin requirement 1 year after diagnosis or a case not meeting the above criteria but in which there had been no insulin requirement for the year after diagnosis	MODY diagnosed through known gene mutation, diabetes as part of a recognized syndrome, as part of a suspected or unrecognized syndrome, or secondary to another condition (e.g., cystic fibrosis).	ICA, GAD, insulin: all negative (only done in unclear cases)	NR	Oral hypoglycemic agent (n=38) (metformin +/- another oral drug [n=34], sulfonylurea +/- another oral drug [n=4]), insulin (n=18), insulin + metformin (n=4), diet alone (n=12)
Shiga, 2009 (Japan) [58]	43	NR	NR	Japan Diabetes Society criteria[59]: fasting plasma glucose ≥7.0 mmol/l or 2-hour plasma glucose ≥11.1 mmol/l in response to OGTT on 2 or more occasions on separate days, or on 1 occasion if subject has typical diabetes symptoms, HbA1c ≥6.5%, or unequivocal diabetic retinopathy.	Secondary or unclassifiable diabetes	GAD and IA-2: all negative	NR	NR

© 2022 Cioana M et al. *JAMA Network Open.*

				Diagnosed based on mode of onset, tendency to ketosis, blood C-peptide value, and negative ICA and GAD antibodies				
Shilbayeh, 2021 (Saudi Arabia)[60]	49	Vitamin D deficiency (n=25), Polydipsia, Polyphagia, and Polyuria (n=23), Acanthosis nigricans (n=19), Hypothyroidism (n=9)	NR	ADA criteria[4]	Older than 19 years, diagnosed with T1DM, and off-label use of metformin	NR	Family history of T2DM (n=42), history of gestational diabetes (n=18)	Insulin + metformin + oral hypoglycemic agent (n=16), metformin + oral hypoglycemic agent (n=11), insulin + metformin (n=10), metformin only (n=9), insulin only (n=3),<br><br>Total number of patients who took oral hypoglycaemic drugs (n=28): DDP-4 inhibitor (n=8), GLP1 agonist (n=6), sulfonylurea + DPP-4 inhibitor (n=5), DPP-4 inhibitor + GLP1 agonist (n=4), sulfonylurea (n=4), TZD (n=2), sulfonylurea + TZD (n=1), sulfonylurea + DPP-4 inhibitor + GLP1 agonist (n=1)
Sugihara, 2005 (Japan) [61]	256	Asymptomatic (most patients)	NR	NR	NR	NR	Family history of diabetes (n=174)	Oral hypoglycemic agent (n=111) (alpha-glucosidase inhibitor [n=61], metformin [n=24], sulfonylurea [n=17], nateglinide [n=5], alpha-glucosidase inhibitor + metformin [n=4]), insulin (n=51), diet + exercise (n=87)
Tung, 2021 (Hong Hong)[62]	391	Hyperglycemia (n=348), Asymptomatic (n=296), Polydipsia (n=81), Polyuria (n=70), Weight loss (n=51), Hyperglycemia + ketosis (n=39), Nocturia (n=27), Lethargy (n=16), Vomiting (n=13), DKA (n=4), Shortness of breath (n=2), Recurrent infection (n=1)	NR	ISPAD guidelines[39]	Patients diagnosed outside the study period or aged out of the pre-defined range	NR	Father alone has T2DM (n=79), mother alone has T2DM (n=99), both parents have T2DM (n=52), siblings alone have T2DM (n=13), at least 1 first degree relative has T2DM (n=150), first- and/or second-degree relatives has T2DM (n=241)	Lifestyle + diet (n=391), oral hypoglycemic agent (n=251), insulin (n=62)
Upchurch, 2003 (USA) [63]	98	Polyuria (n=56), Polydipsia (n=52), Polyuria and Polydipsia (n=46),	NR	NR	NR	ICA: JDF <5 units (n=49/50), unspecified (n=1/50)	Family history of T2DM (n=48)	Oral hypoglycemic agent (n=44), insulin (n=51), insulin + oral hypoglycemic agent (n=13)

© 2022 Cioana M et al. *JAMA Network Open.*

		Polyphagia (n=35), Ketones (n=30), Weight loss (n=29)						
Urakami, 2009 (Japan) [64]	112	NR	NR	Glucose urine test, followed by OGTT to confirm diagnosis	NR	Unspecified: all negative	Family history of T2DM (n>56)	NR
Van Name, 2020 (USA) [65]	998	NR	NR	ADA criteria[4]	NR	NR	NR	Insulin (n=212)
Wei, 2003 (Taiwan) [66]	131	NR	NR	1) fasting plasma glucose ≥7.0 mmol/l at screening and diagnosed as having T2DM by physician. 2) current treatment with an oral hypoglycemic drug or on diet control with no insulin injection; and 3) no recurrent diabetic ketoacidosis	T1DM, drug-induced and unclassified diabetes	NR	Family history of T2DM (n=28)	NR
Xu, 2021 (China)[67]	161	Asymptomatic (n=53)	Tanner I (n=24/101), Tanner II (n=26/101), Tanner III (n=20/101), Tanner IV (n=14/101), Tanner V (n=17/101)	ISPAD guidelines[39]	Positive diabetes-associated autoantibodies (including islet-cell antibody, insulin autoantibody, serum glutamate decarboxylase antibody, protein tyrosine phosphatase antibody, and zinc transporter-8 antibody), inherited disorders, other endocrine diseases, or other serious chronic disorders	ICA: all negative	Family history of T2DM (n=75)	Metformin monotherapy (n=102), insulin (n=NR)
Zabeen, 2016 (Bangladesh) [68]	77	Acanthosis nigricans (n=57), Typical symptoms (n=49), Atypical symptoms (undue fatigability and enuresis, or pruritus vulvae) (n=26), DKA (n=2), Asymptomatic (n=2)	NR	Presence of obesity or overweight, positive family history, presence of acanthosis nigricans, other features of metabolic syndrome, availability of fasting insulin level	NR	None measured	Family history of T2DM (n = 72): 1st-degree relative (n=58), 2nd-degree relative (n=53)	Metformin (n=37), insulin + metformin (n=40)
Zdravkovic, 2004 (Canada) [69]	41	Acanthosis nigricans (n=29), Asymptomatic (n=23), Polyuria, polydipsia and weight loss (n=18), Ketonuria	Pubertal (n=32): early (24%), mid (28%), late puberty (48%)	Presentation and/or clinical course ‘typical’ of T2DM and two or more risk factors for T2DM: obesity defined as BMI >95th percentile, family history of T2DM, race/ethnicity, signs of insulin resistance or	NR	ICA: all negative	Family history of T2DM (n=36), maternal gestational diabetes (n=6)	Oral hypoglycemic agent (metformin or glyburide) (n=7), insulin (n=11), insulin + oral hypoglycemic agent (n=1), diet + exercise (n=22)

© 2022 Cioana M et al. *JAMA Network Open.*

		(n=13), DKA (n=3)		conditions associated with insulin resistance (acanthosis nigricans, polycystic ovarian syndrome, dyslipidemia, hypertension)				
Zuckerman Levin, 2022 (Israel)[70]	379	Acanthosis nigricans (n=188), Asymptomatic (n=160), Polyuria (n=125), Polydipsia (n=118), Striae (n=69), Abdominal obesity (n=58), Weight loss (n=52), Weight gain (n=45), Gastrointestinal symptoms (n=39), Fatigue/dizziness (n=37), Hirsutism (n=34), Headache/visive disturbance (n=28), Nocturia (n=25), Acne (n-17), DKA (n=15), Gynecomastia (n=9)	NR	ADA criteria[4]	Positive islet cell autoantibodies (n=11), genetically confirmed monogenic diabetes, secondary diabetes, and diabetes from various other causes	Unspecified: all negative	Father alone has T2DM (n=141), mother alone has T2DM (n=108), other relative has T2DM (n=184), gestational diabetes (n=27), at least one family member has T2DM (n=277), three generational history of T2DM (n=94)	Metformin alone (n=173), insulin alone (n=79), lifestyle (n=65), metformin + insulin (n=62)

Legend: UK: United Kingdom, USA: United States of America, NR: not reported, DKA: diabetic ketoacidosis, CDA: Canadian Diabetes Association, OGTT: oral glucose tolerance test, T2DM: type 2 diabetes mellitus, ADA: American Diabetes Association, T1DM: type 1 diabetes mellitus, WHO: World Health Organization, ICA: islet cell antibodies, GAD/GAD65: glutamic acid decarboxylase/glutamic acid decarboxylase 65-kilodalton isoform, ISPAD: International Society for Pediatric and Adolescent Diabetes, BMI: body mass index, MODY: maturity onset diabetes of the young, IA-2: islet tyrosine phosphatase 2, JDF: Juvenile Diabetes Foundation, ZnT8: zinc transporter 8, [a]: explanation of including antibody positive patients: Two of the antibody positive patients had elevated serum insulin levels at diagnosis prior to initiation of treatment consistent with β-cell reserve, and one of them was managed with metformin monotherapy for 3 years prior to requiring insulin treatment, which further corroborates T2DM diagnosis. All of the antibody positive patients were obese with acanthosis nigricans and did not present with diabetic ketoacidosis despite medication adherence limitations and elevated HbA1c of ≥9%, [b]: values taken from the whole cohort, not just patients tested for obesity

© 2022 Cioana M et al. *JAMA Network Open.*

eTable 8. Prevalence of Type 2 Diabetes in Patients Without Obesity and Association With Glycemic Control and Dyslipidemia

Author, Year (Country)	Obesity prevalence (n, %)	Non-obese prevalence (n, %)		HbA1c (%) for patients with and without obesity	Dyslipidemia prevalence for patients with and without obesity (n, %)
		Overweight	Normal weight		
Abbasi, 2017 (UK) [2]	308 (47.1)	61 (9.3)	285 (43.6)	NR	NR
Alsaffar, 2020 (Iraq) [3]	16 (100.0)	0 (0.0)	0 (0.0)	9.01± 2.27	DL (unspecified): 3 (18.8)
Amed, 2012 (Canada) [5]	211 (95.5)	10 (4.7)		NR	DL (unspecified): 95 (43.0)
Astudillo, 2021 (USA) [7]	295 (88.6)	33 (9.9)	5 (1.5)	Pubertal: 9.6 ± 2.6 Prepubertal : 8.8 ± 2.2	DL (unspecified): 329 (87.5)
Balasanthiran, 2012 (UK) [8]	23 (59.0)[a]	11 (28.2)[a]	5 (12.8)[a]	8.4 ± 2.35	NR
Bell, 2009 (USA) [9]	83 (79.0)	12 (11.4)	10 (9.5)	<8: 81 (76.4), 8-9.5: 12 (11.3), ≥9.5: 13 (12.3)	High TG: 62 (59.0), High LDL-C: 52 (49.5), Low HDL-C: 66 (62.9)
Campbell-Stokes, 2005 (New Zealand) [11]	11 (92.0)	1 (8.3)		NR	NR
Candler, 2018 (UK and Republic of Ireland) [12]	86 (81.1)	16 (15.1)	4 (3.8)	NR	DL (unspecified): 10 (9.4)
Carino, 2021 (Canada) [13]	232 (72.2)	69 (21.3)	21 (6.5)	9.2 ± 2.6	High LDL-C: 28 (8.6), High TG: 161 (50.0)
Coddington, 2001 (USA) [14]	18 (81.8)	4 (18.2)		NR	NR
Dean, 1992 (Canada) [16]	9 (45.0)	11 (55.0)		NR	NR
Ehtisham, 2004 (UK) [17]	18 (72.0)	5 (20.0)	2 (8.0)	NR	NR
Eppens, 2006 (Australia) [19]	36 (56.3)	16 (25.0)	12 (18.8)	7.3 (6.0–8.3)[c]	High TG: 34 (53.1), High TC: 20 (31.3)
Eppens, 2006 (Western Pacific) [21]	106 (32.0)	103 (31.1)	122 (36.9)	7.0 (5.9–9.9)[c]	High TG: 53 (16.0), High TC: 40 (12.1), High LDL-C: 40 (12.1), Low HDL-C: 33 (10.0)
Farah, 2006 (USA) [22]	29 (72.5)	5 (12.5)	6 (15.0)	9	NR

© 2022 Cioana M et al. *JAMA Network Open.*

Fortmeier-Saucier, 2008 (USA) [23]	44 (89.8)	5 (10.2)		NR	Two or more abnormal lipid values: 37 (75.5)
Fu, 2013 (China) [24]	248 (71.1)	101 (28.9)		NR	High TG: 120 (34.4), High TC: 88 (25.2), Low HDL-C: 109 (31.2)
Glaser, 1998 (USA) [26]	9 (50.0)	9 (50.0)		NR	NR
Greenup, 2020 (USA) [27]	40 (95.2)	2 (4.8)	0 (0.0)	10.5 ± 2.4	High LDL-C: 7 (25.9), Low HDL-C: 14 (51.9)
Grinstein, 2003 (USA) [28]	>70%	<30%		10.9 ± 3.3	NR
Guven, 2016 (Turkey) [29]	53 (63.1)	31 (36.9)		8.4 ± 2.8	High TG: 39 (46.4), High TC: 16 (19.0), High LDL-C: 18 (21.4), Low HDL-C: 46 (54.8)
Haynes, 2014 (Australia) [30]	82 (60.7)	16 (11.9)	36 (26.7)	9.0 ± 2.8	NR
Huang, 2006 (Taiwan) [31]	15 (68.2)	4 (18.2)	3 (13.6)	NR	NR
Kim, 20221 (USA) [32]	237 (80.1)	41 (13.9)	18 (6.1)	8.4 ± 2.7	High LDL-C: 49 (16.4), Low HDL-C: 138 (46.3),High TC: 60 (20.1), High TG: 161 (54.0)
Kitagawa, 1994 (Japan) [33]	111 (85.4)	19 (14.6)		NR	NR
Larkin, 2015 (USA) [34]	605 (88.3)	70 (10.2)	0 (0.0)	NR	NR
Lawrence, 2008 (USA) [35]	401 (77.1)	64 (12.3)	53 (10.2)	NR	NR
Liu, 2009 (USA) [36]	331 (77.2)	50 (11.7)	48 (11.2)	NR	NR
Liu, 2009 (API) (USA) [37]	38 (76.0)	8 (16.0)	4 (8.0)	<8: 24 (55.8), 8-9.5: 3 (7.0), ≥9.5: 16 (37.2)	NR
Ludwig, 2021 (Australia and New Zealand) [38]	199 (76.5)	46 (17.7)	15 (5.8)	7.3 (6.0–9.2)[c]	High TG: 39 (42.9), High LDL: 20 (29.0)
Marks, 2021 (USA)[40]	126 (73.7)	45 (26.3)		pre-COVID-19 pandemic: 9.0 (6.9–11.4)[c], post-COVID-19 pandemic:	NR

© 2022 Cioana M et al. *JAMA Network Open.*

				10.2 (7.9–11.3)[c]	
Morrison, 2018 (UK) [41]	9 (50.0)	7 (38.9)	2 (11.1)	8.4 (6.5 - 11.2)	NR
Newton, 2015 (New Zealand) [42]	22 (95.7)	1 (4.3)		NR	NR
Osman, 2013 (Sudan) [43]	29 (76.3)	8 (21.1)	1 (2.6)	9.1	DL (unspecified): 6 (15.8)
Pérez-Perdomo, 2005 (Puerto Rico) [44]	69 (80.2)	8 (9.3)	9 (10.5)	<6: 7 (15.6), 6-7: 1 (24.4), >7: 27 (60.0)	High TG: 31 (36.1), High TC: 51 (59.2)
Pinhas-Hamiel, 1996 (USA) [45]	50 (92.6)	4 (7.4)		NR	High TG: 2 (3.7)
Ramachandran, 2003 (India) [46]	9 (50.0)	6 (33.3)	3 (16.7)	10.35	High TG: 5 (27.8), High TC: 3 (16.7), Combined DL: 2 (11.1)
Reinehr, 2005 (Germany) [47]	14 (87.5)	2 (12.5)	0 (0.0)	6.9 (5.8 - 8.5)[c]	High TG: 9 (56.3), High TC: 4 (25.0), High LDL-C: 4 (25.0), Low HDL-C: 8 (50.0)
Reinehr, 2008 (Germany and Austria) [48]	85 (65.9)	34 (26.4)	10 (7.8)	7.4 (6.0–9.1)[c]	DL (unspecified): 84 (65.1)
Ruhayel, 2010 (Australia) [50]	23 (69.7)	4 (12.1)	6 (18.2)	6.5 (4.5–15.7)[d]	DL (unspecified): 18 (54.5)
Schmitt, 2022 (USA)[52]	474 (77.1)	141 (22.9)		8.1 (6.5-11.7)[c]	NR
Scott, 1997 (USA) [53]	42 (85.7)	5 (10.2)	2 (4.1)	11 ± 0.6[b]	NR
Scott, 2004 (New Zealand) [54]	13 (100.0)	0 (0.0)		8.8	High TG: 1 (7.7), High LDL-C: 4 (30.8)
Sellers, 2007 (Canada) [55]	38 (38.4)	43 (43.4)	18 (18.2)	>7: 58 (58.6), <7: 41 (41.4)	High TG: 59 (59.6), High TC: 43 (43.4), High LDL-C: 41 (41.4), Low HDL-C: 35 (35.4)
Shield, 2009 (UK and Republic of Ireland) [57]	61 (80.3)	5 (6.6)	3 (3.9)	NR	NR
Shiga, 2009 (Japan) [58]	28 (65.1)	15 (34.9)		6.9 ± 1.7 (4.9–12.6)	NR
Shilbayeh, 2021 (Saudi Arabia)[60]	38 (77.6)	11 (22.4)		9.4	DL (unspecified): 6 (12.2)
Sugihara, 2005 (Japan) [61]	179 (69.9)	78 (30.1)		8.8 ± 2.8	NR

© 2022 Cioana M et al. *JAMA Network Open.*

Tung, 2021 (Hong Hong)[62]	308 (78.7)	58 (14.8)	25 (6.5)	NR	DL (unspecified): 138 (35.3)
Upchurch, 2003 (USA) [63]	91 (92.9)	4 (4.1)	3 (3.0)	10.38 ± 3.52	NR
Urakami, 2009 (Japan) [64]	93 (83.0)	19 (17.0)		9.6 ± 2.6	High TG: 37 (33.0), Low HDL-C: 24 (21.4)
Van Name, 2020 (USA) [65]	909 (91.1)	77 (7.7)	12 (1.2)	10.0 (7.5–12.2)[c]	NR
Wei, 2003 (Taiwan) [66]	63 (48.1)	68 (51.9)		NR	High TC: 35 (26.7)
Xu, 2021 (China)[67]	89 (58.2)	19 (12.4)	45 (29.4)	11.5 (8.9-13)[c]	DL (unspecified): 81 (53.2)
Zabeen, 2016 (Bangladesh) [68]	45 (58.4)	25 (32.5)	7 (9.1)	10.6 ± 2.7	High TG: 10 (13.0), High TC: 27 (35.1), Combined DL: 5 (6.5)
Zdravkovic, 2004 (Canada) [69]	33 (80.5)	8 (19.5)		10 ± 3.4	High TG and High TC: 16 (39.0)
Zuckerman Levin, 2022 (Israel)[70]	278 (77.2)	82 (22.8)		8.8 ± 2.5	High TG: 173 (45.6), low HDL-C: 214 (56.5)

Legend: NR: not reported, TG: triglycerides, TC: total cholesterol, LDL-C: low-density lipoprotein cholesterol, HDL-C: high-density lipoprotein cholesterol, DL: dyslipidemia; [a]: estimated based on graph; HbA1c reported as mean, mean (range), mean ± standard deviation, range: n(%), [b]: mean ± standard error, [c]: median (interquartile range), [d]: median (range)

© 2022 Cioana M et al. *JAMA Network Open.*

eTable 9. Results of Sensitivity Analyses

Studies removed	Pooled prevalence estimate (%, 95% CI)	I^2, χ^2 p-value
**Conference abstracts** [29,30,41,42]	75.75% (95% CI 70.78-80.40)	95%, p-value < 0.001
**Sample size <50** [3,8,11,14,16,17,22,23,26,27,31,41–43,46,47,50,53,54,58,60,69]	74.01% (95% CI 67.78-79.90)	97%, p-value < 0.001
**Patients over 18 years old** [2,8,14,22,23,40,43,46,54,60,65]	75.09% (95% CI 70.17-79.71)	95%, p-value < 0.001
**Different obesity definition** [3,5,8,22,26,29,30,33,41,45–47,52,58,61,62,64]	75.23% (95% CI 68-94-81.05)	97%, p-value < 0.001
**Inclusion criteria of overweight** [34,47,65]	74.25% (95% CI 69.49-78.76)	95%, p-value < 0.001
**Diabetes diagnosis criteria unspecified/unclear** [8,16,22,23,29,30,32,41,42,44,61,63]	75.71% (95% CI 70.06-80.96)	97%, p-value < 0.001
**Inclusion of patients with weight loss at presentation** [8,12,14,22,27,32,40,43,45,53,62,63,69,70]	72.87% (95% CI 66.58-78.75)	97%, p-value < 0.001
**Inclusion of patients with positive pancreatic autoantibodies** [11,16,17,27,29,63]	74.81% (95% CI 69.72-79.59)	96%, p-value < 0.001
**No mention of specifically excluding MODY**[8,12,17,27,35,38,40,45,48,57,70]	78.87% (95% CI 74.70-82.77)	85%, p-value < 0.001
**Non-population based studies**[2,5,11–13,17,21,24,33–35,38,44,48,57,60–62,65–67,70]	74.43% (95% CI 66.53-81.62)	98%, p-value < 0.001

© 2022 Cioana M et al. *JAMA Network Open.*

eTable 10. Risk of Bias of Included Studies
Scores: 0: no; 1: yes; overall risk of bias: low (score >8), moderate (score 6-8), or high (score ≤5).
Items scored: 1) was the study's target population a close representation of the national population in relation to relevant variables (e.g., age, sex)?; 2) was the sampling frame a true or close representation of the target population?; 3) was some form of random selection used to select the sample, OR, was a census undertaken?; 4) was the likelihood of non-response bias minimal?; 5) were data collected directly from the subjects (as opposed to a proxy)?; 6) was an acceptable case definition used in the study?; 7) had the study instrument that measured the parameter of interest (e.g., prevalence of comorbidity) been tested for reliability and validity (if necessary)?; 8) was the same mode of data collection used for all subjects?; 9) was the length of the shortest prevalence period for the parameter of interest appropriate?; 10) were the numerator(s) and denominator(s) for the parameter of interest appropriate?

	**External Validity Items**				**Internal Validity Items**						**Overall Score**	**Overall Risk of Bias**
**Author, year (country)**	**1**	**2**	**3**	**4**	**5**	**6**	**7**	**8**	**9**	**10**		
Abbasi, 2017 (UK) [2]	1	1	1	1	1	1	1	0	1	1	9	low
Alsaffar, 2020 (Iraq) [3]	0	1	1	1	1	1	1	1	1	1	9	low
Amed, 2012 (Canada) [5]	1	0	0	1	1	1	1	0	1	1	7	moderate
Astudillo, 2021 (USA) [7]	0	1	1	1	1	1	1	1	1	1	9	low
Balasanthiran, 2012 (UK) [8]	0	0	1	1	1	1	1	1	1	1	8	moderate
Bell, 2009 (USA) [9]	0	1	1	1	1	1	1	1	1	1	9	low
Campbell-Stokes, 2005 (New Zealand) [11]	1	1	1	1	1	1	1	0	1	1	9	low
Candler, 2018 (UK and Republic of Ireland) [12]	1	1	1	1	1	1	1	0	1	1	9	low
Carino, 2021 (Canada) [13]	1	1	1	1	1	1	1	1	1	1	10	low
Coddington, 2001 (USA) [14]	0	1	1	1	1	1	1	1	1	1	9	low
Dean, 1992 (Canada) [16]	0	1	1	1	1	1	1	1	1	1	9	low
Ehtisham, 2004 (UK) [17]	1	1	1	1	1	1	1	1	1	1	10	low
Eppens, 2006 (Australia) [19]	0	1	1	0	1	1	1	1	1	1	8	moderate
Eppens, 2006 (Western Pacific) [21]	1	0	1	1	1	1	1	1	1	1	9	low
Farah, 2006 (USA) [22]	0	0	0	1	1	1	1	1	1	0	6	moderate
Fortmeier-Saucier, 2008 (USA) [23]	0	0	0	1	1	1	1	1	1	1	7	moderate
Fu, 2013 (China) [24]	1	1	1	1	1	1	1	1	1	1	10	low
Glaser, 1998 (USA) [26]	0	1	1	1	1	1	1	0	1	1	8	moderate
Greenup, 2020 (USA) [27]	0	0	0	1	1	1	1	0	1	1	6	moderate
Grinstein, 2003 (USA) [28]	0	0	0	0	1	1	1	1	1	1	6	moderate
Guven, 2016 (Turkey) [29]	0	0	0	1	1	1	1	1	1	1	7	moderate
Haynes, 2014 (Australia) [30]	0	1	1	0	1	1	1	1	1	1	8	moderate

© 2022 Cioana M et al. *JAMA Network Open.*

Huang, 2006 (Taiwan) [31]	0	1	1	1	1	1	1	1	1	1	9	low
Kim, 20221 (USA) [32]	0	1	1	1	1	1	1	1	1	1	9	low
Kitagawa, 1994 (Japan) [33]	1	0	1	1	1	1	1	1	1	1	9	low
Larkin, 2015 (USA) [34]	1	1	0	1	1	1	1	1	1	1	9	low
Lawrence, 2008 (USA) [35]	1	1	1	1	1	1	1	1	1	1	10	low
Liu, 2009 (USA) [36]	1	1	1	1	1	1	1	1	1	1	10	low
Liu, 2009 (API) (USA) [37]	0	1	1	0	1	1	1	1	1	1	8	moderate
Ludwig, 2021 (Australia and New Zealand) [38]	1	1	1	0	1	1	1	1	1	1	9	low
Marks, 2021 (USA)[40]	0	1	1	0	1	1	1	1	1	1	8	moderate
Morrison, 2018 (UK) [41]	0	0	0	1	1	1	1	1	1	1	7	moderate
Newton, 2015 (New Zealand) [42]	0	1	1	0	1	1	1	1	1	1	8	moderate
Osman, 2013 (Sudan) [43]	0	1	1	1	1	1	1	1	1	1	9	low
Pérez-Perdomo, 2005 (Puerto Rico) [44]	1	0	1	0	1	1	1	0	1	1	7	moderate
Pinhas-Hamiel, 1996 (USA) [45]	0	1	1	1	1	1	1	1	1	1	9	low
Ramachandran, 2003 (India) [46]	0	0	1	1	1	1	1	1	1	1	8	moderate
Reinehr, 2005 (Germany) [47]	0	1	1	0	1	1	1	1	1	1	8	moderate
Reinehr, 2008 (Germany and Austria) [48]	1	1	1	0	1	1	1	0	1	1	8	moderate
Ruhayel, 2010 (Australia) [50]	0	1	1	0	1	1	1	1	1	1	8	moderate
Schmitt, 2022 (USA)[52]	0	1	1	1	1	1	1	1	1	1	9	low
Scott, 1997 (USA) [53]	0	0	0	1	1	1	1	1	1	1	7	moderate
Scott, 2004 (New Zealand) [54]	0	1	1	1	1	1	1	0	1	1	8	moderate
Sellers, 2007 (Canada) [55]	0	1	1	1	1	1	1	1	1	1	9	low
Shield, 2009 (UK and Republic of Ireland) [57]	1	1	1	1	1	1	1	0	1	1	9	low
Shiga, 2009 (Japan) [58]	0	0	0	1	1	1	1	1	1	1	7	moderate
Shilbayeh, 2021 (Saudi Arabia)[60]	1	1	1	0	1	1	1	1	1	1	9	low
Sugihara, 2005 (Japan) [61]	1	1	1	0	1	1	1	1	1	1	9	low
Tung, 2021 (Hong Kong)[62]	1	1	1	1	1	1	1	1	1	1	10	low
Upchurch, 2003 (USA) [63]	0	0	0	0	1	1	1	1	1	1	6	moderate
Urakami, 2009 (Japan) [64]	0	0	0	1	1	1	1	1	1	1	7	moderate
Van Name, 2020 (USA) [65]	1	1	0	1	1	1	1	0	1	1	8	moderate
Wei, 2003 (Taiwan) [66]	1	1	1	1	1	1	1	1	1	1	10	low

© 2022 Cioana M et al. *JAMA Network Open.*

Xu, 2021 (China)[67]	1	1	1	0	1	1	1	1	1	1	9	low
Zabeen, 2016 (Bangladesh)[68]	0	1	1	1	1	1	1	1	1	1	9	low
Zdravkovic, 2004 (Canada)[69]	0	1	1	1	1	1	1	1	1	1	9	low
Zuckerman Levin, 2022 (Israel)[70]	1	1	1	1	1	1	1	1	1	1	10	low

© 2022 Cioana M et al. *JAMA Network Open.*

eFigure 1. Study Flow Diagram

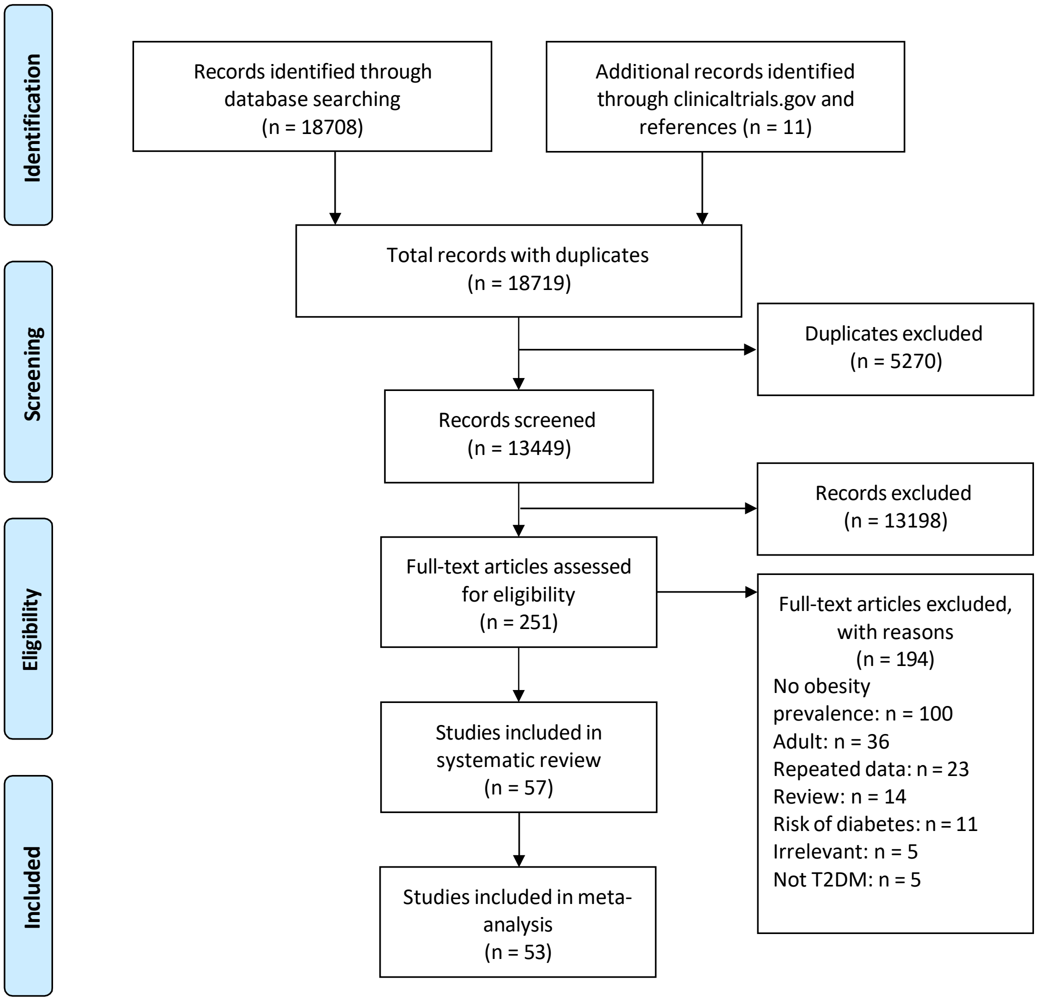

© 2022 Cioana M et al. *JAMA Network Open.*

eFigure 2. Forest Plot Illustrating Odds Ratio of Obesity in Pediatric T2DM by Sex

	Males		Females			Odds Ratio	Odds Ratio
Study or Subgroup	Events	Total	Events	Total	Weight	IV, Random, 95% CI	IV, Random, 95% CI
Alsaffar, 2020 (Iraq) (61)	10	10	6	6		Not estimable	
Glaser, 1998 (USA) (41)	5	6	4	12	3.0%	10.00 [0.85, 117.02]	
Guven, 2016 (Turkey) (91)	21	26	32	58	9.9%	3.41 [1.13, 10.29]	
Kitagawa, 1994 (Japan) (38)	56	58	55	72	6.6%	8.65 [1.91, 39.24]	
Lawrence, 2008 (USA) (51)	147	193	254	327	19.7%	0.92 [0.60, 1.40]	
Ramachandran, 2003 (India) (42)	3	5	6	13	3.9%	1.75 [0.22, 14.22]	
Ruhayel, 2010 (Australia) (89)	9	11	14	22	5.2%	2.57 [0.44, 14.97]	
Sellers, 2007 (Canada) (71)	19	42	19	57	13.4%	1.65 [0.73, 3.75]	
Shiga, 2009 (Japan) (55)	13	15	15	28	5.7%	5.63 [1.07, 29.74]	
Sugihara, 2005 (Japan) (70)	93	119	86	137	17.5%	2.12 [1.22, 3.70]	
Wei, 2003 (Taiwan) (4)	27	50	36	81	15.1%	1.47 [0.72, 2.98]	
**Total (95% CI)**		**535**		**813**	**100.0%**	**2.10 [1.33, 3.31]**	
Total events	403		527				

Heterogeneity: $Tau^2 = 0.23$; $Chi^2 = 18.94$, df = 9 (P = 0.03); $I^2 = 52\%$

Test for overall effect: Z = 3.19 (P = 0.001)

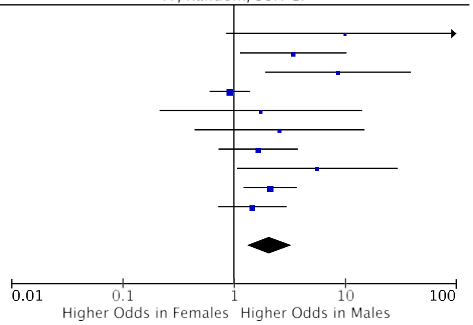

eFigure 3. Forest Plot Illustrating Prevalence of Obesity in Pediatric T2DM by Region

Study	Cases	Total	Weight	Prevalence (%) [95% CI]
**Region = Americas**				
Dean, 1992 (Canada) (64)	9	20	1.6%	45.00 [23.57; 67.36]
Pinhas−Hamiel, 1996 (USA) (39)	50	54	1.9%	92.59 [83.80; 98.35]
Scott, 1997 (USA) (40)	42	49	1.9%	85.71 [74.33; 94.33]
Glaser, 1998 (USA) (41)	9	18	1.5%	50.00 [26.81; 73.19]
Coddington, 2001 (USA) (65)	18	22	1.6%	81.82 [62.53; 95.64]
Upchurch, 2003 (USA) (43)	91	98	2.0%	92.86 [86.79; 97.25]
Zdravkovic, 2004 (Canada) (67)	33	41	1.8%	80.49 [66.77; 91.39]
Pérez−Perdomo, 2005 (Puerto Rico) (69)	69	86	2.0%	80.23 [71.08; 88.04]
Farah, 2006 (USA) (49)	29	40	1.8%	72.50 [57.49; 85.39]
Sellers, 2007 (Canada) (71)	38	99	2.0%	38.38 [29.01; 48.20]
Fortmeier−Saucier, 2008 (USA) (59)	44	49	1.9%	89.80 [79.50; 96.99]
Lawrence, 2008 (USA) (51)	401	520	2.1%	77.12 [73.40; 80.63]
Amed, 2012 (Canada) (57)	211	221	2.1%	95.48 [92.28; 97.88]
Larkin, 2015 (USA) (90)	605	685	2.1%	88.32 [85.80; 90.62]
Greenup, 2020 (USA) (80)	40	42	1.8%	95.24 [86.18; 99.92]
Van Name, 2020 (USA) (79)	909	998	2.1%	91.08 [89.23; 92.78]
Astudillo, 2021 (USA) (81)	295	333	2.1%	88.59 [84.94; 91.80]
Carino, 2021 (Canada) (92)	232	322	2.1%	72.05 [67.01; 76.83]
Marks, 2021 (USA) (82)	126	171	2.1%	73.68 [66.80; 80.03]
Kim, 2022 (USA) (84)	237	296	2.1%	80.07 [75.31; 84.43]
Schmitt, 2022 (USA) (85)	474	615	2.1%	77.07 [73.66; 80.31]
**Total (95% CI)**		**4779**	**40.6%**	**81.14 [75.99; 85.83]**
Heterogeneity: $Tau^2 = 0.0173$; $Chi^2 = 314.19$, df = 20 ($P < 0.01$); $I^2 = 94\%$				
**Region = Middle East**				
Osman, 2013 (Sudan) (74)	29	38	1.8%	76.32 [61.34; 88.68]
Guven, 2016 (Turkey) (91)	53	84	2.0%	63.10 [52.46; 73.14]
Alsaffar, 2020 (Iraq) (61)	16	16	1.5%	100.00 [89.52; 100.00]
Shilbayeh, 2021 (Saudi Arabia) (62)	38	49	1.9%	77.55 [64.69; 88.29]
Zuckerman Levin, 2022 (Israel) (86)	278	360	2.1%	77.22 [72.74; 81.42]
**Total (95% CI)**		**547**	**9.2%**	**78.41 [68.29; 87.12]**
Heterogeneity: $Tau^2 = 0.0113$; $Chi^2 = 17.17$, df = 4 ($P < 0.01$); $I^2 = 77\%$				
**Region = Oceania**				
Scott, 2004 (New Zealand) (68)	13	13	1.4%	100.00 [87.18; 100.00]
Campbell−Stokes, 2005 (New Zealand) (46)	11	12	1.3%	91.67 [67.61; 100.00]
Eppens, 2006 (Australia) (11)	36	64	1.9%	56.25 [43.89; 68.24]
Eppens, 2006 (Western Pacific) (48)	106	331	2.1%	32.02 [27.10; 37.16]
Ruhayel, 2010 (Australia) (89)	23	33	1.8%	69.70 [52.78; 84.37]
Haynes, 2014 (Australia) (75)	82	135	2.0%	60.74 [52.34; 68.84]
Newton, 2015 (New Zealand) (76)	22	23	1.6%	95.65 [82.27; 100.00]
Ludwig, 2021 (Australia and New Zealand) (60)	199	260	2.1%	76.54 [71.18; 81.50]
**Total (95% CI)**		**871**	**14.2%**	**74.03 [55.52; 89.15]**
Heterogeneity: $Tau^2 = 0.0667$; $Chi^2 = 178.85$, df = 7 ($P < 0.01$); $I^2 = 96\%$				
**Region = Asia**				
Kitagawa, 1994 (Japan) (38)	111	130	2.0%	85.38 [78.74; 90.99]
Ramachandran, 2003 (India) (42)	9	18	1.5%	50.00 [26.81; 73.19]
Wei, 2003 (Taiwan) (4)	63	131	2.0%	48.09 [39.56; 56.68]
Sugihara, 2005 (Japan) (70)	179	256	2.1%	69.92 [64.15; 75.40]
Huang, 2006 (Taiwan) (50)	15	22	1.6%	68.18 [47.00; 86.28]
Shiga, 2009 (Japan) (55)	28	43	1.8%	65.12 [50.15; 78.76]
Urakami, 2009 (Japan) (56)	93	112	2.0%	83.04 [75.47; 89.48]
Fu, 2013 (China) (73)	248	349	2.1%	71.06 [66.18; 75.71]
Zabeen, 2016 (Bangladesh) (58)	45	77	2.0%	58.44 [47.21; 69.26]
Tung, 2021 (Hong Kong) (83)	308	391	2.1%	78.77 [74.57; 82.69]
Xu, 2021 (China) (63)	89	153	2.0%	58.17 [50.24; 65.89]
**Total (95% CI)**		**1682**	**21.3%**	**68.54 [61.15; 75.51]**
Heterogeneity: $Tau^2 = 0.0134$; $Chi^2 = 87.27$, df = 10 ($P < 0.01$); $I^2 = 89\%$				
**Region = Europe**				
Ehtisham, 2004 (UK) (45)	18	25	1.7%	72.00 [52.55; 88.15]
Reinehr, 2005 (Germany) (47)	14	16	1.5%	87.50 [65.90; 99.73]
Reinehr, 2008 (Germany and Austria) (87)	85	129	2.0%	65.89 [57.46; 73.85]
Shield, 2009 (UK and Republic of Ireland) (88)	61	76	2.0%	80.26 [70.48; 88.53]
Balasanthiran, 2012 (UK) (72)	23	39	1.8%	58.97 [43.05; 74.03]
Abbasi, 2017 (UK) (77)	308	654	2.1%	47.09 [43.28; 50.93]
Candler, 2018 (UK and Republic of Ireland) (3)	86	106	2.0%	81.13 [73.07; 88.07]
Morrison, 2018 (UK) (78)	9	18	1.5%	50.00 [26.81; 73.19]
**Total (95% CI)**		**1063**	**14.6%**	**68.30 [54.84; 80.43]**
Heterogeneity: $Tau^2 = 0.0321$; $Chi^2 = 87.94$, df = 7 ($P < 0.01$); $I^2 = 92\%$				
**Total (95% CI)**		**8942**	**100.0%**	**75.27 [70.47; 79.78]**
Heterogeneity: $Tau^2 = 0.0336$; $Chi^2 = 1199.39$, df = 52 ($P < 0.01$); $I^2 = 96\%$				

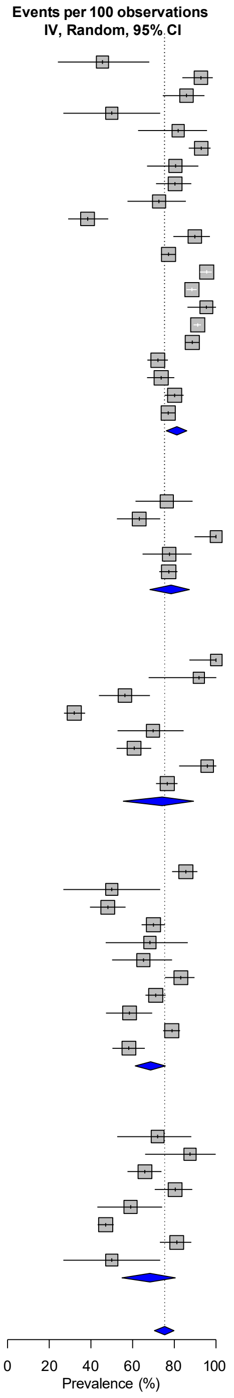

© 2022 Cioana M et al. *JAMA Network Open.*

eFigure 4. Forest Plot Illustrating Prevalence of Normal BMI-Based Measures in Pediatric T2DM by Region

Study	Cases	Total	Weight	Prevalence (%) [95% CI]
**Region = Europe**				
Abbasi, 2017 (UK) (77)	285	654	3.3%	43.58 [39.80; 47.40]
Balasanthiran, 2012 (UK) (72)	5	39	2.9%	12.82 [3.84; 25.43]
Candler, 2018 (UK and Republic of Ireland) (3)	4	106	3.2%	3.77 [0.82; 8.42]
Ehtisham, 2004 (UK) (45)	2	25	2.8%	8.00 [0.15; 22.62]
Morrison, 2018 (UK) (78)	2	18	2.6%	11.11 [0.23; 30.65]
Reinehr, 2005 (Germany) (47)	0	16	2.5%	0.00 [0.00; 10.48]
Reinehr, 2008 (Germany and Austria) (87)	10	129	3.2%	7.75 [3.68; 13.08]
Shield, 2009 (UK and Republic of Ireland) (88)	3	76	3.1%	3.95 [0.50; 9.76]
**Total (95% CI)**		**1063**	**23.6%**	**9.52 [0.46; 25.83]**
Heterogeneity: $Tau^2 = 0.0850$; $Chi^2 = 221.55$, df = 7 ($P < 0.01$); $I^2 = 97\%$				
**Region = Middle East**				
Alsaffar, 2020 (Iraq) (61)	0	16	2.5%	0.00 [0.00; 10.48]
Osman, 2013 (Sudan) (74)	1	38	2.9%	2.63 [0.00; 10.95]
**Total (95% CI)**		**54**	**5.5%**	**1.26 [0.00; 7.32]**
Heterogeneity: $Tau^2 = 0$; $Chi^2 = 0.24$, df = 1 ($P = 0.62$); $I^2 = 0\%$				
**Region = Americas**				
Astudillo, 2021 (USA) (81)	5	333	3.3%	1.50 [0.42; 3.14]
Carino, 2021 (Canada) (92)	21	322	3.3%	6.52 [4.06; 9.50]
Farah, 2006 (USA) (49)	6	40	2.9%	15.00 [5.34; 27.98]
Greenup, 2020 (USA) (80)	0	42	3.0%	0.00 [0.00; 4.05]
Kim, 2022 (USA) (84)	18	296	3.3%	6.08 [3.61; 9.12]
Larkin, 2015 (USA) (90)	0	685	3.3%	0.00 [0.00; 0.25]
Lawrence, 2008 (USA) (51)	53	520	3.3%	10.19 [7.73; 12.95]
Pérez−Perdomo, 2005 (Puerto Rico) (69)	9	86	3.1%	10.47 [4.75; 17.93]
Scott, 1997 (USA) (40)	0	49	3.0%	0.00 [0.00; 3.48]
Sellers, 2007 (Canada) (71)	18	99	3.2%	18.18 [11.14; 26.45]
Upchurch, 2003 (USA) (43)	3	98	3.1%	3.06 [0.38; 7.62]
Van Name, 2020 (USA) (79)	12	998	3.3%	1.20 [0.61; 1.99]
**Total (95% CI)**		**3568**	**38.0%**	**4.21 [1.55; 7.93]**
Heterogeneity: $Tau^2 = 0.0158$; $Chi^2 = 200.06$, df = 11 ($P < 0.01$); $I^2 = 95\%$				
**Region = Oceania**				
Eppens, 2006 (Australia) (11)	12	64	3.1%	18.75 [10.01; 29.34]
Eppens, 2006 (Western Pacific) (48)	122	331	3.3%	36.86 [31.73; 42.13]
Haynes, 2014 (Australia) (75)	36	135	3.2%	26.67 [19.52; 34.48]
Ludwig, 2021 (Australia and New Zealand) (60)	15	260	3.2%	5.77 [3.22; 8.97]
Ruhayel, 2010 (Australia) (89)	6	33	2.9%	18.18 [6.58; 33.43]
Scott, 2004 (New Zealand) (68)	0	13	2.4%	0.00 [0.00; 12.82]
**Total (95% CI)**		**836**	**18.1%**	**16.43 [5.37; 31.51]**
Heterogeneity: $Tau^2 = 0.0420$; $Chi^2 = 105.53$, df = 5 ($P < 0.01$); $I^2 = 95\%$				
**Region = Asia**				
Huang, 2006 (Taiwan) (50)	3	22	2.7%	13.64 [1.91; 31.71]
Ramachandran, 2003 (India) (42)	3	18	2.6%	16.67 [2.40; 38.00]
Tung, 2021 (Hong Kong) (83)	25	391	3.3%	6.39 [4.16; 9.05]
Xu, 2021 (China) (63)	45	153	3.2%	29.41 [22.43; 36.90]
Zabeen, 2016 (Bangladesh) (58)	7	77	3.1%	9.09 [3.52; 16.69]
**Total (95% CI)**		**661**	**14.9%**	**13.95 [4.52; 26.93]**
Heterogeneity: $Tau^2 = 0.0268$; $Chi^2 = 45.5$, df = 4 ($P < 0.01$); $I^2 = 91\%$				
**Total (95% CI)**		**6182**	**100.0%**	**8.27 [4.16; 13.45]**
Heterogeneity: $Tau^2 = 0.0500$; $Chi^2 = 1177.05$, df = 32 ($P < 0.01$); $I^2 = 97\%$				

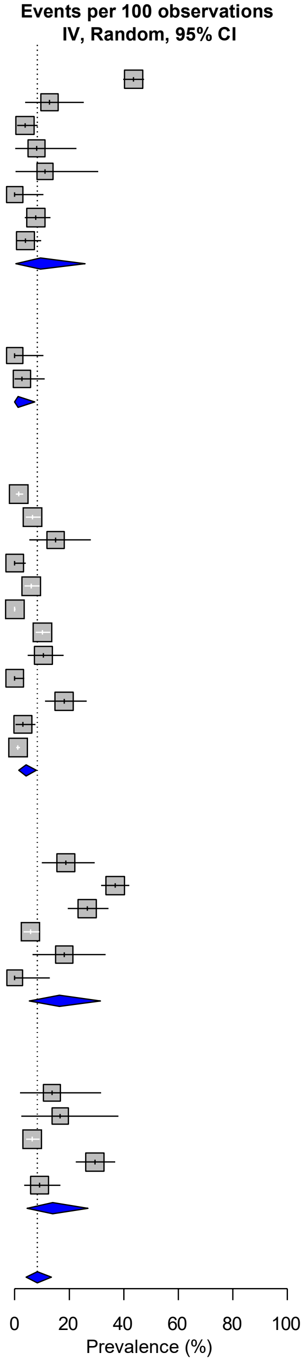

© 2022 Cioana M et al. *JAMA Network Open.*

eFigure 5. Funnel Plot for Publication Bias for Pooled Prevalence of Obesity in Pediatric Type 2 Diabetes

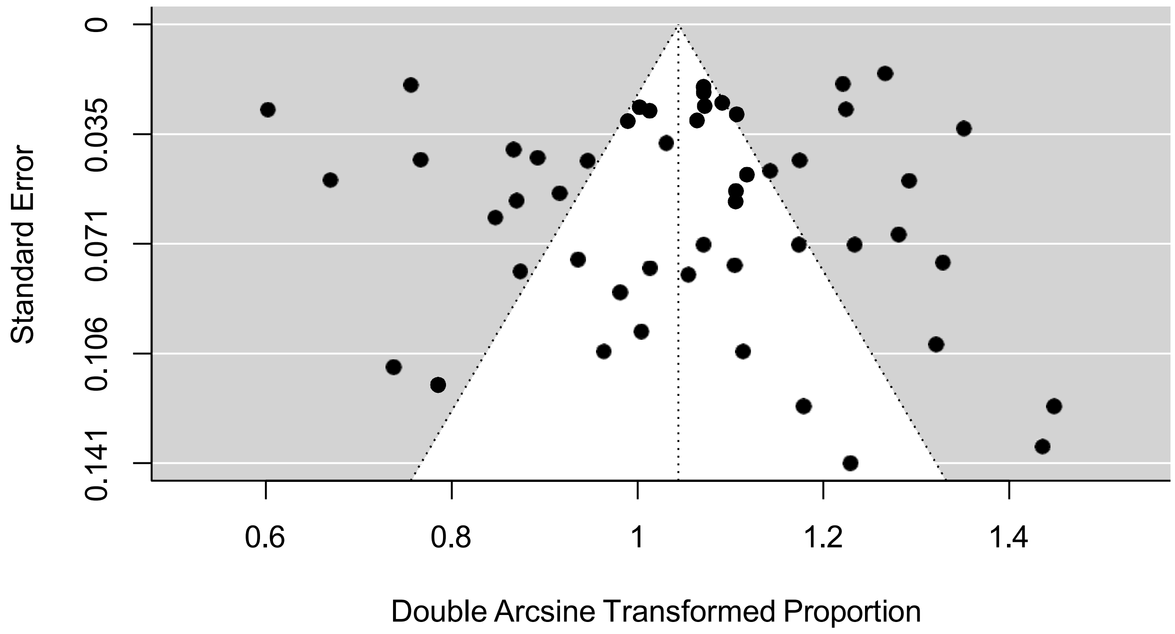

© 2022 Cioana M et al. *JAMA Network Open.*

eFigure 6. Funnel Plot for Publication Bias for Pooled Prevalence of Obesity at Type 2 Diabetes Diagnosis in Patients With Pediatric Type 2 Diabetes

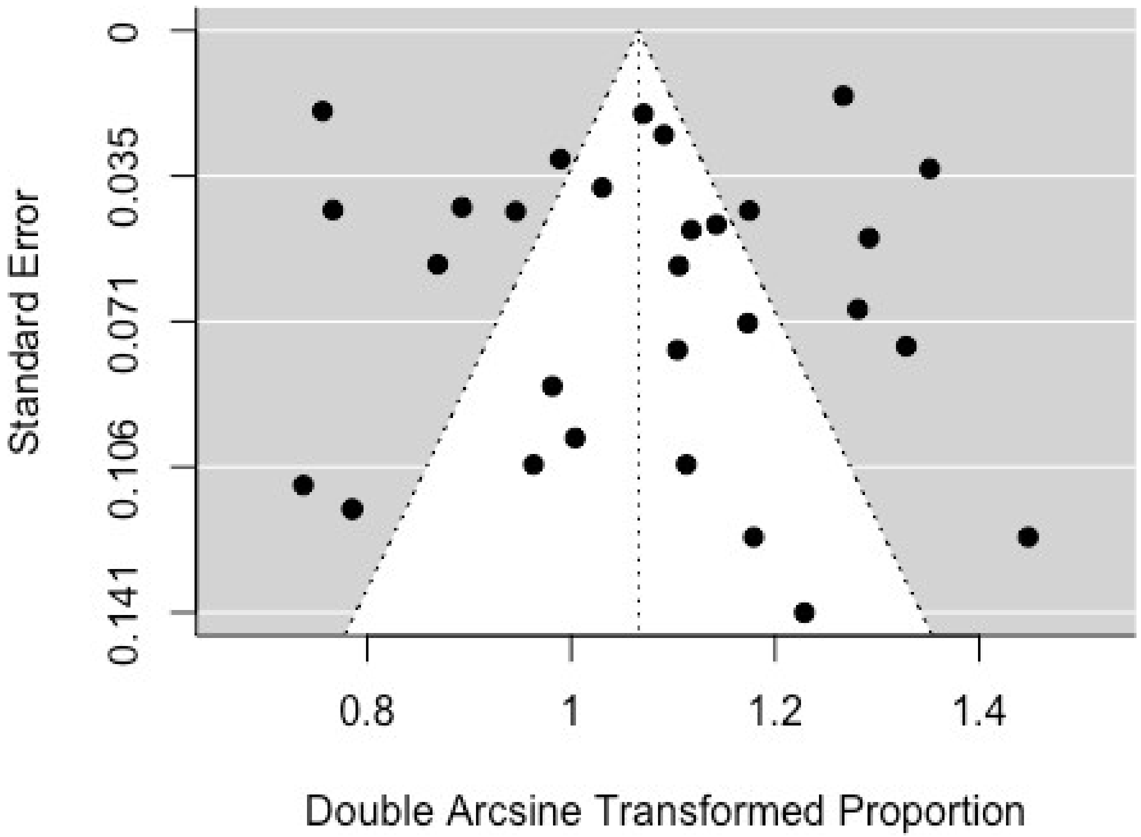

© 2022 Cioana M et al. *JAMA Network Open.*

eReferences

1. Stroup DF, Berlin JA, Morton SC, et al. Meta-analysis of Observational Studies in Epidemiology: A Proposal for Reporting. *JAMA*. 2000;283(15):2008-2012. doi:10.1001/jama.283.15.2008

2. Abbasi A, Juszczyk D, van Jaarsveld CHM, Gulliford MC. Body Mass Index and Incident Type 1 and Type 2 Diabetes in Children and Young Adults: A Retrospective Cohort Study. *J Endocr Soc*. 2017;1(5):524-537. doi:10.1210/js.2017-00044

3. Alsaffar Y, Hussain AM, Selman NA. Prevalence of Type 2 Diabetes in pediatrics and adolescents newly diagnosed with diabetes in Babylon Governorate, Iraq. *Archivos Venezolanos de Farmacología y Terapéutica*. 2020;39(7):839-843.

4. American Diabetes Association. Type 2 Diabetes in Children and Adolescents. *Pediatrics*. 2000;105(3):671-680. doi:10.1542/peds.105.3.671

5. Amed S, Hamilton JK, Sellers EAC, et al. Differing clinical features in Aboriginal vs. non-Aboriginal children presenting with type 2 diabetes. *Pediatric Diabetes*. 2012;13(6):470-475. doi:10.1111/j.1399-5448.2012.00859.x

6. Canadian Diabetes Association. Clinical practice guidelines for the prevention and management of diabetes in Canada. *Canadian Journal of Diabetes*. 2008;32.

7. Astudillo M, Tosur M, Castillo B, et al. Type 2 diabetes in prepubertal children. *Pediatric Diabetes*. 2021;22(7):946-950. doi:10.1111/pedi.13254

8. Balasanthiran A, O'Shea T, Moodambail A, et al. Type 2 diabetes in children and young adults in East London: an alarmingly high prevalence. *Practical Diabetes*. 2012;29(5):193-198a. doi:10.1002/pdi.1689

9. Bell RA, Mayer-Davis EJ, Beyer JW, et al. Diabetes in Non-Hispanic White Youth. *Diabetes Care*. 2009;32(Suppl 2):S102-S111. doi:10.2337/dc09-S202

10. American Diabetes Association. Report of the Expert Committee on the diagnosis and classification of diabetes Mellitus. *Diabetes Care*. 2000;23:S4-S19.

11. Campbell-Stokes PL, Taylor BJ, on behalf of the New Zealand Children's Diabetes Working Group. Prospective incidence study of diabetes mellitus in New Zealand children aged 0 to 14 years. *Diabetologia*. 2005;48(4):643-648. doi:10.1007/s00125-005-1697-3

12. Candler TP, Mahmoud O, Lynn RM, Majbar AA, Barrett TG, Shield JPH. Continuing rise of Type 2 diabetes incidence in children and young people in the UK. *Diabetic Medicine*. 2018;35(6):737-744. doi:10.1111/dme.13609

13. Carino M, Elia Y, Sellers E, et al. Comparison of Clinical and Social Characteristics of Canadian Youth Living With Type 1 and Type 2 Diabetes. *Canadian Journal of Diabetes*. 2021;45(5):428-435. doi:10.1016/j.jcjd.2021.01.008

© 2022 Cioana M et al. *JAMA Network Open.*

14. Coddington DA, Hisnanick JJ. Clinical Characteristics of Non-insulin-dependent Diabetes Mellitus among Southwestern American Indian Youths. *Journal of Health, Population and Nutrition*. 2001;19(1):12-17.

15. National Diabetes Data Group. Classification and diagnosis of diabetes mellitus and other categories of glucose intolerance. *Diabetes*. 1979;28(12):1039-1057. doi:10.2337/diab.28.12.1039

16. Dean HJ, Mundy RL, Moffatt M. Non-insulin-dependent diabetes mellitus in Indian children in Manitoba. *CMAJ*. 1992;147(1):52-57.

17. Ehtisham S, Hattersley AT, Dunger DB, Barrett TG. First UK survey of paediatric type 2 diabetes and MODY. *Archives of Disease in Childhood*. 2004;89(6):526-529. doi:10.1136/adc.2003.027821

18. World Health Organization. Definition, diagnosis and classification of diabetes mellitus and its complications : report of a WHO consultation. Part 1, Diagnosis and classification of diabetes mellitus. Published online 1999. Accessed May 21, 2021. https://apps.who.int/iris/handle/10665/66040

19. Eppens MC, Craig ME, Cusumano J, et al. Prevalence of diabetes complications in adolescents with type 2 compared with type 1 diabetes. *Diabetes Care*. 2006;29(6):1300-1306. doi:10.2337/dc05-2470

20. Australasian Paediatric Endocrine Group, Department of Health and Ageing. Clinical Practice Guidelines: Type 1 Diabetes In Children and Adolescents: (509522012-001). Published online 2004. doi:10.1037/e509522012-001

21. Eppens MC, Craig ME, Jones TW, et al. Type 2 diabetes in youth from the Western Pacific region: glycaemic control, diabetes care and complications. *Current Medical Research and Opinion*. 2006;22(5):1013-1020. doi:10.1185/030079906X104795

22. Farah SE, Wals KT, Friedman IB, Pisacano MA, DiMartino-Nardi J. Prevalence of Retinopathy and Microalbuminuria in Pediatric Type 2 Diabetes Mellitus. *Journal of Pediatric Endocrinology and Metabolism*. 2006;19(7). doi:10.1515/JPEM.2006.19.7.937

23. Fortmeier-Saucier Linda, Savrin Carol, Heinzer Marjorie, Hudak Christine. BMI and Lipid Levels in Mexican American Children Diagnosed with Type 2 Diabetes. *Worldviews on Evidence-Based Nursing*. 2008;5(3):142-147. doi:10.1111/j.1741-6787.2008.00122.x

24. Fu JF, Liang L, Gong CX, et al. Status and trends of diabetes in Chinese children: analysis of data from 14 medical centers. *World J Pediatr*. 2013;9(2):127-134. doi:10.1007/s12519-013-0414-4

25. American Diabetes Association. Diagnosis and Classification of Diabetes Mellitus. *Diabetes Care*. 2011;34(Supplement 1):S62-S69. doi:10.2337/dc11-S062

© 2022 Cioana M et al. *JAMA Network Open.*

26. Glaser NS, Jones KL. Non-insulin dependent diabetes mellitus in Mexican-American children. *West J Med*. 1998;168(1):11-16.

27. Greenup E, Sunil B, Barr MM, Ashraf AP. Glycaemic control and outcomes in children with type 2 diabetes diagnosed at or before 10 years of age. *Endocrinology, Diabetes & Metabolism*. n/a(n/a):e00192. doi:https://doi.org/10.1002/edm2.192

28. Grinstein G, Muzumdar R, Aponte L, Vuguin P, Saenger P, DiMartino-Nardi J. Presentation and 5-Year Follow-Up of Type 2 Diabetes mellitus in African-American and Caribbean-Hispanic Adolescents. *Hormone Research in Paediatrics*. 2003;60(3):121-126. doi:10.1159/000072523

29. Guven A, Demir Gokce E. Cardiovascular Risk and Long Term Follow-up of Turkish Children with Type 2 Diabetes: Single Center Experience. *Horm Res Paediatr*. 2016;86(Suppl 1):244.

30. Haynes A, Kalic R, Curran J, et al. Type 2 diabetes and associated complications in Western Australian children: a population-based study (1990-2012). *Diabetologia*. 2014;57(Suppl 1):S510-S510.

31. Huang CY, Li HJ, Lo FS, et al. Metabolic Disorders in Children and Adolescents with Type 2 Diabetes Mellitus. *Acta Paediatr Taiwan*. 2006;47(4):187-191.

32. Kim G, DeSalvo D, Guffey D, et al. Dyslipidemia in adolescents and young adults with type 1 and type 2 diabetes: a retrospective analysis. *International Journal of Pediatric Endocrinology*. 2020;2020(1):NA-NA. doi:10.1186/s13633-020-00081-7

33. Kitagawa T, Owada M, Urakami T, Tajima N. Epidemiology of type 1 (insulin-dependent) and type 2 (non-insulin-dependent) diabetes mellitus in Japanese children. *Diabetes Research and Clinical Practice*. 1994;24:S7-S13. doi:10.1016/0168-8227(94)90221-6

34. Larkin ME, Walders-Abramson N, Hirst K, et al. Effects of comorbid conditions on health-related quality of life in youth with Type 2 diabetes: the TODAY clinical trial. *Diabetes Manag (Lond)*. 2015;5(6):431-439. doi:10.2217/dmt.15.35

35. Lawrence JM, Liese AD, Liu L, et al. Weight-Loss Practices and Weight-Related Issues Among Youth With Type 1 or Type 2 Diabetes. *Diabetes Care*. 2008;31(12):2251-2257. doi:10.2337/dc08-0719

36. Liu LL, Lawrence JM, Davis C, et al. Prevalence of overweight and obesity in youth with diabetes in USA: the SEARCH for Diabetes in Youth Study. *Pediatric Diabetes*. 2010;11(1):4-11. doi:10.1111/j.1399-5448.2009.00519.x

37. Liu LL, Yi JP, Beyer J, et al. Type 1 and Type 2 Diabetes in Asian and Pacific Islander U.S. Youth. *Diabetes Care*. 2009;32(Suppl 2):S133-S140. doi:10.2337/dc09-S205

38. Ludwig K, Craig ME, Donaghue KC, Maguire A, Benitez-Aguirre PZ. Type 2 diabetes in children and adolescents across Australia and New Zealand: A 6-year audit from The

© 2022 Cioana M et al. *JAMA Network Open.*

Australasian Diabetes Data Network (ADDN). *Pediatric Diabetes*. 2021;22(3):380-387. doi:10.1111/pedi.13169

39. Zeitler P, Arslanian S, Fu J, et al. ISPAD Clinical Practice Consensus Guidelines 2018: Type 2 diabetes mellitus in youth. *Pediatric Diabetes*. 2018;19(S27):28-46. doi:10.1111/pedi.12719

40. Marks BE, Khilnani A, Meyers A, et al. Increase in the Diagnosis and Severity of Presentation of Pediatric Type 1 and Type 2 Diabetes during the COVID-19 Pandemic. *Horm Res Paediatr*. 2021;94(7-8):275-284. doi:10.1159/000519797

41. Morrison A, Chatterjee S, Greening J, et al. Phenotype and burden of comorbidities in adolescents with Type 2 diabetes in a multiethnic population. *Diabetic Medicine*. 2018;35:107. doi:10.1111/dme.29_13571

42. Newton K, Stanley J, Wiltshire E. Audit of type 2 diabetes in youth in Wellington, New Zealand 2001–2013. *Pediatric Diabetes*. 2015;16(Suppl 21):50-150. doi:10.1111/pedi.12309

43. Osman HAM, Elsadek N, Abdullah MA. Type 2 diabetes in Sudanese children and adolescents. *Sudan J Paediatr*. 2013;13(2):17-23.

44. Pérez-Perdomo R, Pérez-Cardona CM, Allende-Vigo M, Rivera-Rodríguez MI, Rodríguez-Lugo LA. Type 2 diabetes mellitus among youth in Puerto Rico, 2003. *P R Health Sci J*. 2005;24(2):111-117.

45. Pinhas-Hamiel O, Dolan LM, Daniels SR, Standiford D, Khoury PR, Zeitler P. Increased incidence of non-insulin-dependent diabetes mellitus among adolescents. *The Journal of Pediatrics*. 1996;128(5):608-615. doi:10.1016/S0022-3476(96)80124-7

46. Ramachandran A, Snehalatha C, Satyavani K, Sivasankari S, Vijay V. Type 2 Diabetes in Asian-Indian Urban Children. *Diabetes Care*. 2003;26(4):1022-1025. doi:10.2337/diacare.26.4.1022

47. Reinehr T, Andler W, Kapellen T, et al. Clinical Characteristics of Type 2 Diabetes Mellitus in Overweight European Caucasian Adolescents. *Exp Clin Endocrinol Diabetes*. 2005;113(03):167-170. doi:10.1055/s-2005-837522

48. Reinehr T, Schober E, Roth CL, Wiegand S, Holl R. Type 2 Diabetes in Children and Adolescents in a 2-Year Follow-Up: Insufficient Adherence to Diabetes Centers. *Hormone Research in Paediatrics*. 2008;69(2):107-113. doi:10.1159/000111814

49. International, Society for Pediatric and Adolescent Diabetes. Consensus Guidelines of the International Society for Pediatric and Adolescent Diabetes. Published online 2000. http://www.d4pro.com/diabetesguidelines/

50. Ruhayel SD, James RA, Ehtisham S, Cameron FJ, Werther GA, Sabin MA. An observational study of type 2 diabetes within a large Australian tertiary hospital pediatric

diabetes service. *Pediatric Diabetes*. 2010;11(8):544-551. doi:10.1111/j.1399-5448.2010.00647.x

51. Rosenbloom AL, Silverstein JH, Amemiya S, Zeitler P, Klingensmith GJ, International Society for Pediatric and Adolescent Diabetes. ISPAD Clinical Practice Consensus Guidelines 2006-2007. Type 2 diabetes mellitus in the child and adolescent. *Pediatr Diabetes*. 2008;9(5):512-526. doi:10.1111/j.1399-5448.2008.00429.x

52. Schmitt JA, Ashraf AP, Becker DJ, Sen B. Changes in Type 2 Diabetes Trends in Children and Adolescents During the COVID-19 Pandemic. *J Clin Endocrinol Metab*. 2022;107(7):e2777-e2782. doi:10.1210/clinem/dgac209

53. Scott CR, Smith JM, Cradock MM, Pihoker C. Characteristics of Youth-onset Noninsulin-dependent Diabetes Mellitus and Insulin-dependent Diabetes Mellitus at Diagnosis. *Pediatrics*. 1997;100(1):84-91. doi:10.1542/peds.100.1.84

54. Scott A, Whitcombe S, Bouchier D, Dunn P. Diabetes in children and young adults in Waikato Province, New Zealand: outcomes of care. *NZMJ*. 2004;117(1207).

55. Sellers EA, Yung G, Dean HJ. Dyslipidemia and other cardiovascular risk factors in a Canadian First Nation pediatric population with type 2 diabetes mellitus. *Pediatric Diabetes*. 2007;8(6):384-390. doi:10.1111/j.1399-5448.2007.00284.x

56. Canadian Diabetes Association Clinical Practice Guideline Expert Committee. Canadian Diabetes Association 2003 clinical practice guidelines for the prevention and management of diabetes in Canada. *Canadian Journal of Diabetes*. 2003;27(Suppl. 2):S1-S153.

57. Shield JPH, Lynn R, Wan KC, Haines L, Barrett TG. Management and 1 year outcome for UK children with type 2 diabetes. *Archives of Disease in Childhood*. 2009;94(3):206-209. doi:10.1136/adc.2008.143313

58. Shiga K, Kikuchi N. Children with type 2 diabetes mellitus are at greater risk of macrovascular complications. *Pediatrics International*. 2009;51(4):563-567. doi:10.1111/j.1442-200X.2009.02836.x

59. Kuzuya T, Nakagawa S, Satoh J, et al. Report of the Committee on the classification and diagnostic criteria of diabetes mellitus. *Diabetes Res Clin Pract*. 2002;55(1):65-85. doi:10.1016/s0168-8227(01)00365-5

60. Shilbayeh S. Type 2 diabetes mellitus and its effect on quality of life in adolescents: A retrospective cohort study in Saudi Arabia. *Pediatr Endocrinol Diabetes Me*. 2022;28(1):54-63. doi:10.5114/pedm.2022.113988

61. Sugihara S, Sasaki N, Kohno H, Amemiya S, Tanaka T, Matsuura N. Survey of Current Medical Treatments for Childhood-Onset Type 2 Diabetes Mellitus in Japan. *Clin Pediatr Endocrinol*. 2005;14(2):65-75. doi:10.1297/cpe.14.65

© 2022 Cioana M et al. *JAMA Network Open.*

62. Tung JY ling, Kwan EY wah, But BW man, et al. Incidence and clinical characteristics of pediatric-onset type 2 diabetes in Hong Kong: The Hong Kong childhood diabetes registry 2008 to 2017. *Pediatric Diabetes*. 2021;n/a(n/a):1-6. doi:10.1111/pedi.13231

63. Upchurch SL, Brosnan CA, Meininger JC, et al. Characteristics of 98 Children and Adolescents Diagnosed With Type 2 Diabetes by Their Health Care Provider at Initial Presentation. *Diabetes Care*. 2003;26(7):2209-2209. doi:10.2337/diacare.26.7.2209

64. Urakami T, Suzuki J, Yoshida A, et al. Prevalence of components of the metabolic syndrome in schoolchildren with newly diagnosed type 2 diabetes mellitus. *Pediatric Diabetes*. 2009;10(8):508-512. doi:10.1111/j.1399-5448.2009.00533.x

65. Van Name MA, Cheng P, Gal RL, et al. Children and adolescents with type 1 and type 2 diabetes mellitus in the Pediatric Diabetes Consortium Registries: comparing clinical characteristics and glycaemic control. *Diabetic Medicine*. 2020;37(5):863-867. doi:10.1111/dme.14233

66. Wei JN, Sung FC, Lin CC, Lin RS, Chiang CC, Chuang LM. National Surveillance for Type 2 Diabetes Mellitus in Taiwanese Children. *JAMA*. 2003;290(10):1345-1350. doi:10.1001/jama.290.10.1345

67. Xu ZR, Du HW, Cui LW, et al. Association of β-cell function and insulin resistance with pediatric type 2 diabetes among Chinese children. *World J Diabetes*. 2021;12(8):1292-1303. doi:10.4239/wjd.v12.i8.1292

68. Zabeen B, Nahar J, Tayyeb S, Mohsin F, Nahar N, Azad K. Characteristics of children and adolescents at onset of type 2 diabetes in a Tertiary Hospital in Bangladesh. *Indian J Endocrinol Metab*. 2016;20(5):638-642. doi:10.4103/2230-8210.190544

69. Zdravkovic V, Daneman D, Hamilton J. Presentation and course of Type 2 diabetes in youth in a large multi-ethnic city. *Diabetic Medicine*. 2004;21(10):1144-1148. doi:10.1111/j.1464-5491.2004.01297.x

70. Zuckerman Levin N, Cohen M, Phillip M, et al. Youth-onset Type 2 diabetes in Israel: A national cohort. *Pediatric Diabetes*. 2022;n/a(n/a). doi:10.1111/pedi.13351

© 2022 Cioana M et al. *JAMA Network Open.*
