# Supplementary material for: The Prevalence of Obesity Among Children With Type 2 Diabetes: A Systematic Review and Meta-analysis
Source: JAMA Netw Open. 2022 Dec 15;5(12):e2247186. doi: 10.1001/jamanetworkopen.2022.47186 (PMC9856349; doi:10.1001/jamanetworkopen.2022.47186)
Supplement: Supplement 2. — Data Sharing Statement [file jamanetwopen-e2247186-s002.pdf]

Data Sharing Statement

Cioana. The Prevalence of Obesity Among Children With Type 2 Diabetes. *JAMA Netw Open*. Published December 15, 2022. doi:10.1001/jamanetworkopen.2022.47186

Data
Data available: Yes
Data types: Deidentified participant data
How to access data: all data is already available in the tables provided
When available: With publication

Supporting Documents
Document types: None

Additional Information
Who can access the data: anyone requesting the data
Types of analyses: all analyses
Mechanisms of data availability: n/a
